# Gut microbiota is associated with obesity and cardiometabolic disease in a population in the midst of Westernization

**DOI:** 10.1038/s41598-018-29687-x

**Published:** 2018-07-27

**Authors:** Jacobo de la Cuesta-Zuluaga, Vanessa Corrales-Agudelo, Eliana P. Velásquez-Mejía, Jenny A. Carmona, José M. Abad, Juan S. Escobar

**Affiliations:** 1 Vidarium—Nutrition, Health and Wellness Research Center, Grupo Empresarial Nutresa, Calle 8 sur 50-67, 050023 Medellin, Colombia; 2 Dinámica IPS—Especialista en Ayudas Diagnósticas, Calle 27 45-109, 050021 Medellin, Colombia; 3 EPS SURA, Calle 49A 63-55, 050034 Medellin, Colombia; 4 Present Address: Max Planck Institute for Developmental Biology—Max-Planck-Ring 5, 72076 Tübingen, Germany; 5 Present Address: SURA Colombia, Medellin, Colombia

## Abstract

Westernization and its accompanying epidemiological transitions are associated with changes in gut microbiota. While the extremes of this lifestyle spectrum have been compared (hunter-gatherers, industrialized countries), populations undergoing such shifts have received little attention. To fill the gap of knowledge about the microbiome evolution following broad lifestyle changes and the emergence of disease-associated dysbiosis, we performed a cross-sectional study in which we characterized the microbiota of 441 Colombian adults through 16S rRNA gene sequencing and determined its relationship with demographic, health-related and dietary parameters. We showed that in the gut microbiota of this cohort thrive taxa proper of both hunter-gatherers (*Prevotella*, *Treponema*) and citizens of industrialized countries (*Bacteroides*, *Bifidobacterium*, *Barnesiella*); the relative abundances of these taxa differed from those in Western and non-Western populations. We also showed that the Colombian gut microbiota is composed of five consortia of co-abundant microorganisms that are differentially associated with lifestyle, obesity and cardiometabolic disease, and highlighted metabolic pathways that might explain associations between microbiota and host health. Our results give insights into the evolution of the gut microbiota, and underscore the importance of this community to human health. Promoting the growth of specific microbial consortia could help ameliorating physiological conditions associated with Western lifestyles.

## Introduction

The gut microbiota is fundamental to human health^1^, and its modulation may prove pivotal to the future of personalized medicine and nutrition^2^. However, identifying the ways in which this microbial community associates with health is not straightforward because the gut microbiota is diverse, complex^3^, and varies according to geographic origin and lifestyle of the host^4–7^. The latter is especially relevant in the context of Westernization^8^, a growing nutritional and epidemiological transition characterized by changes in diet, reduced physical activity and increased prevalence of non-communicable diseases^9^.

Most comparative studies have contrasted the gut microbiota of extremely different populations, usually hunter-gatherers and urban inhabitants of industrialized countries. Hunter-gatherers harbor highly diverse gut microbiota rich in fiber-degrading organisms^4–7^, whereas Westernized microbiota have depleted diversity and higher levels of potentially pathogenic microbes^3,8^. Nevertheless, little attention has been given to populations in the midst of Westernization, that is, populations with recent nutritional and epidemiological shifts, making it unclear whether the microbiota evolves gradually along this lifestyle spectrum or whether there is a breaking point compelling this community to adopt a Westernized configuration.

Colombians are a good model to understand the changes associated with Westernization. A traditional diet rich in complex carbohydrates, mainly rice, potato and corn^10^ (Table S1), suggests that the nutritional transition in this population progresses at a slow pace; in contrast, the epidemiological transition is *en route*, as reflected in recent economic growth, a rapid shift from rural to urban settings^11^, and increasing incidences of physical inactivity^12^ and non-communicable diseases, particularly obesity and cardiovascular disease^13^. Furthermore, Colombians harbor a gut microbiota distinct from that of populations from industrialized countries^14^ and a different genetic background^15^. Considering this combination of Western lifestyle and non-Western diet, in addition to particular ancestral genetics^16^, we hypothesized that the composition of the gut microbiota of Colombians shares taxa proper of both ends of the lifestyle spectrum, making it possible to recognize Western and non-Western microbial configurations associated with host health. In agreement with this hypothesis, we show that Colombians harbor a gut microbiota that cannot be classified as Western or non-Western, composed of five consortia of co-abundant microorganisms (CAGs)—which are phylogenetically and/or functionally related—that exhibit contrasting associations with obesity and cardiometabolic risk factors. Our results are important for understanding the emergence of associations between lifestyle-driven dysbiosis and disease risk in the context of broad lifestyle changes.

## Results

### The colombian gut microbiota is neither traditional nor western

We enrolled 441 community-dwelling participants (18–62 years old, with body mass index—BMI ≥ 18.5 kg/m^2^) from the five largest urban centers in Colombia (Bogota, Medellin, Cali, Barranquilla and Bucaramanga, that make up to 30% of the Colombian population) who donated stool samples for 16S rRNA gene sequencing and thoroughly assessed their demographic, health-related and dietary parameters. Participants were enrolled in roughly similar proportions by the city of origin, sex, age range (18–40 and 41–62 years) and BMI (lean, overweight and obese) (Table S2). After bioinformatic curation of the DNA sequences, 14,750,448 reads passed the quality filters and were grouped into 4,720 operational taxonomic units (OTUs) delimited at 97% identity.

**Figure 1:**
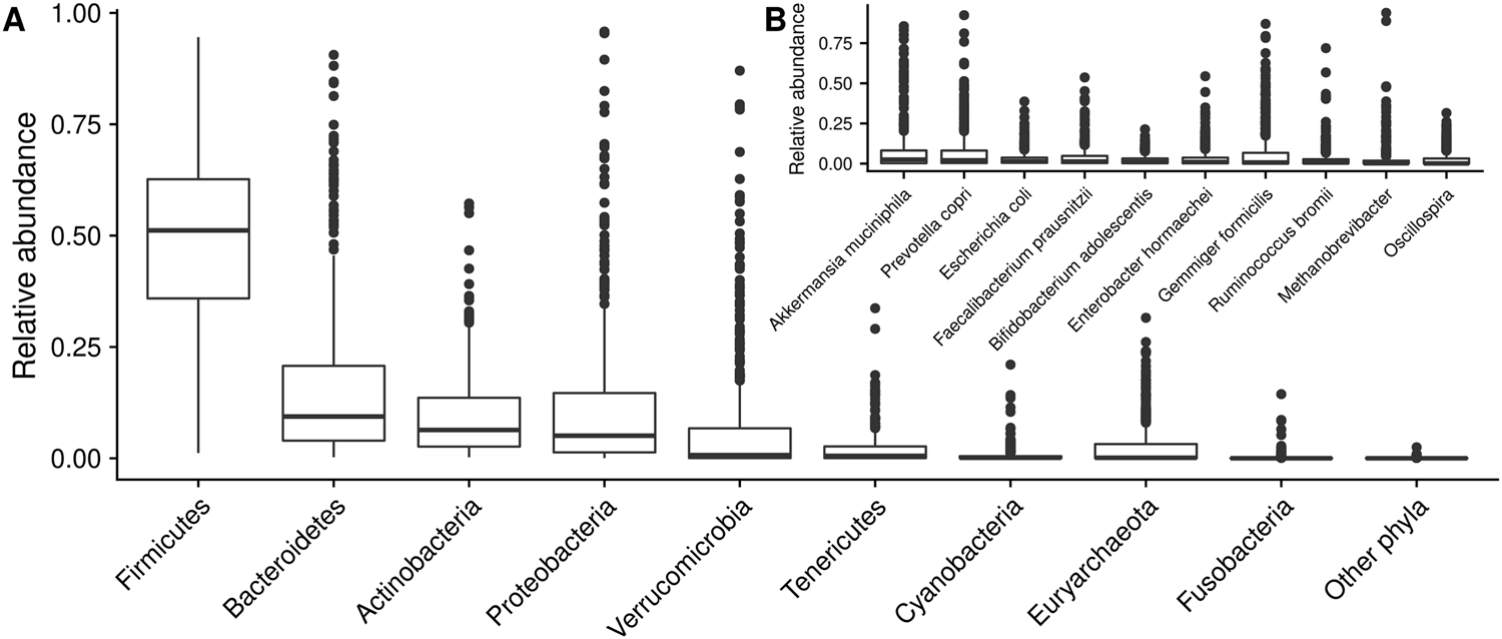
Taxonomic profiles of the gut microbiota of Colombians. **(A)** Relative abundance at the phylum level. Phyla with a median abundance equal to zero were combined into “other phyla”. **(B)** Relative abundance of the 10 OTUs with the highest mean abundance.

Firmicutes, Bacteroidetes and Actinobacteria dominated the gut microbiota of Colombians. Remarkably, the abundances of Proteobacteria and Verrucomicrobia were highly variable, ranging from <1% up to 96% and 87%, respectively (Fig. 1A). This translated into high but uneven levels of OTUs classified as *Akkermansia muciniphila*, *Prevotella copri*, *Escherichia coli*, *Faecalibacterium prausnitzii*, *Bifidobacterium adolescentis*, *Enterobacter hormaechei*, *Gemmiger formicilis*, *Ruminococcus bromii*, *Methanobrevibacter* and *Oscillospira* (Fig. 1B).

**Figure 2:**
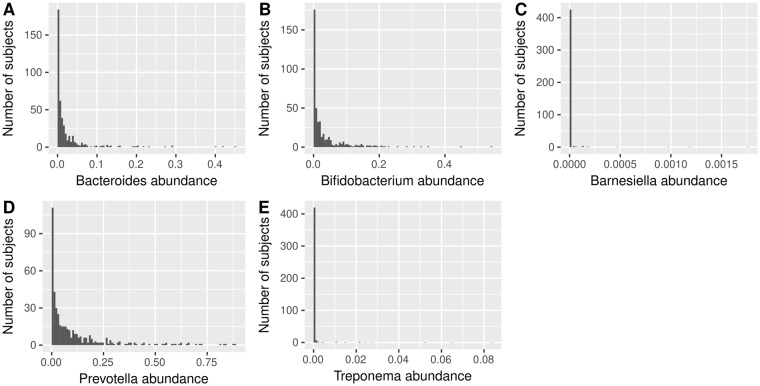
Abundances of marker taxa of Western and non-Western gut microbiota extracted from previous studies^4,17^ and confirmed by the analysis of public benchmark datasets. **(A)** *Bacteroides*, **(B)** *Bifidobacterium*, **(C)** *Barnesiella*, **(D)** *Prevotella*, and **(E)** *Treponema*. Note the differences of scale between plots.

Multiple studies have demonstrated marked differences in the composition of gut microbiota between industrialized and rural populations^4–7^, and recent meta-analyses have provided a list of marker taxa for distinguishing between Western and non-Western microbial communities (*Prevotella* and *Treponema* for non-Westerners; *Bacteroides*, *Bifidobacterium* and *Barnesiella* for Westerners)^17^. We corroborated that these marker taxa had different abundances between Western and non-Western microbiota through the analysis of 16 benchmark datasets analyzed with curatedMetagenomicData^18^, including 1655 subjects from 16 countries (Table S3). In Colombians, all OTUs classified as *Prevotella* (174 OTUs) had a mean (±SD) abundance of 10.7 ± 16.2% (t test for the null hypothesis that the mean abundance was not significantly greater than zero: p < 0.0001), while *Bacteroides* (101 OTUs) and *Bifidobacterium* (15 OTUs) had mean abundance of 2.5 ± 5.3% (t test: p < 0.0001) and 3.5 ± 6.3% (t test: p < 0.0001), respectively. Furthermore, we detected positive abundances of the hunter-gatherer-associated *Treponema* (14 OTUs; 0.08 ± 0.6%; t test: p = 0.002), and of Western-associated *Barnesiella* (10 OTUs; 0.001 ± 0.011%; t test: p = 0.01) (Fig. 2). The relative abundances of lifestyle markers in the Colombian cohort was significantly different to those in the benchmark datasets (all t tests: p < 0.01). Together, these results suggest that Colombians harbor a non-traditional, non-Western gut microbiota, rich in fiber-degrading microbes proper of non-Western communities and, simultaneously, microbes typically found in Western communities.

### The gut microbiota of Colombians does not cluster into enterotypes

We next evaluated whether the non-traditional, non-Western gut microbiota of Colombians structured into discrete microbial configurations or enterotypes^19^. We aimed to determine whether part of the individuals of our cohort harbored a type of microbiota (*e*.*g*., non-Western) and other individuals different types (*e*.*g*., Western). Alternatively, each individual could have a microbiota in which both Western and non-Western microbes thrived. To this end, we used the OTU-level abundance profile, calculated the square root of the Jensen-Shannon divergence and the weighted UniFrac distance to obtain distance matrices which were then used to cluster samples with the partitioning around medoids (PAM) algorithm^19,20^. Then, calculated the average silhouette index (SI) for clusters between 2 to 20, taking 0.50 and 0.75 as thresholds for moderate and strong clustering, respectively. We found poor support for the existence of discrete microbiota clusters (SI < 0.2 in all cases). Furthermore, the *Prevotella*-*Bacteroides* co-exclusion did not differentiate types of microbiota in Colombians (Supplementary Results).

To corroborate this, we selected OTUs with a median abundance ≥0.01% across participants and tested their correlations with the first three axes of the principal coordinate analysis (PCoA) of weighted UniFrac distances (which together accounted for more than 40% of the total variance), using Spearman’s correlation coefficients and FDR-adjusted p-values. We retained a total of 100 OTUs, which collectively represented 80.0 ± 12.5% of the total 16S rRNA reads. These OTUs included Western and non-Western marker taxa as well as other organisms not considered lifestyle biomarkers. We found more OTUs associated with these three PCoA axes than expected under an enterotype configuration (14 OTUs significantly correlated with PCo1, 13 with PCo2 and 11 with PCo3; Table S4), demonstrating that the gut microbiota of Colombians has a complex multispecies nature and is better described by an enterogradient (*i*.*e*., a continuum of abundances of microbial taxa).

### Consortia of related microorganisms are useful for describing the gut microbiota of Colombians

**Figure 3:**
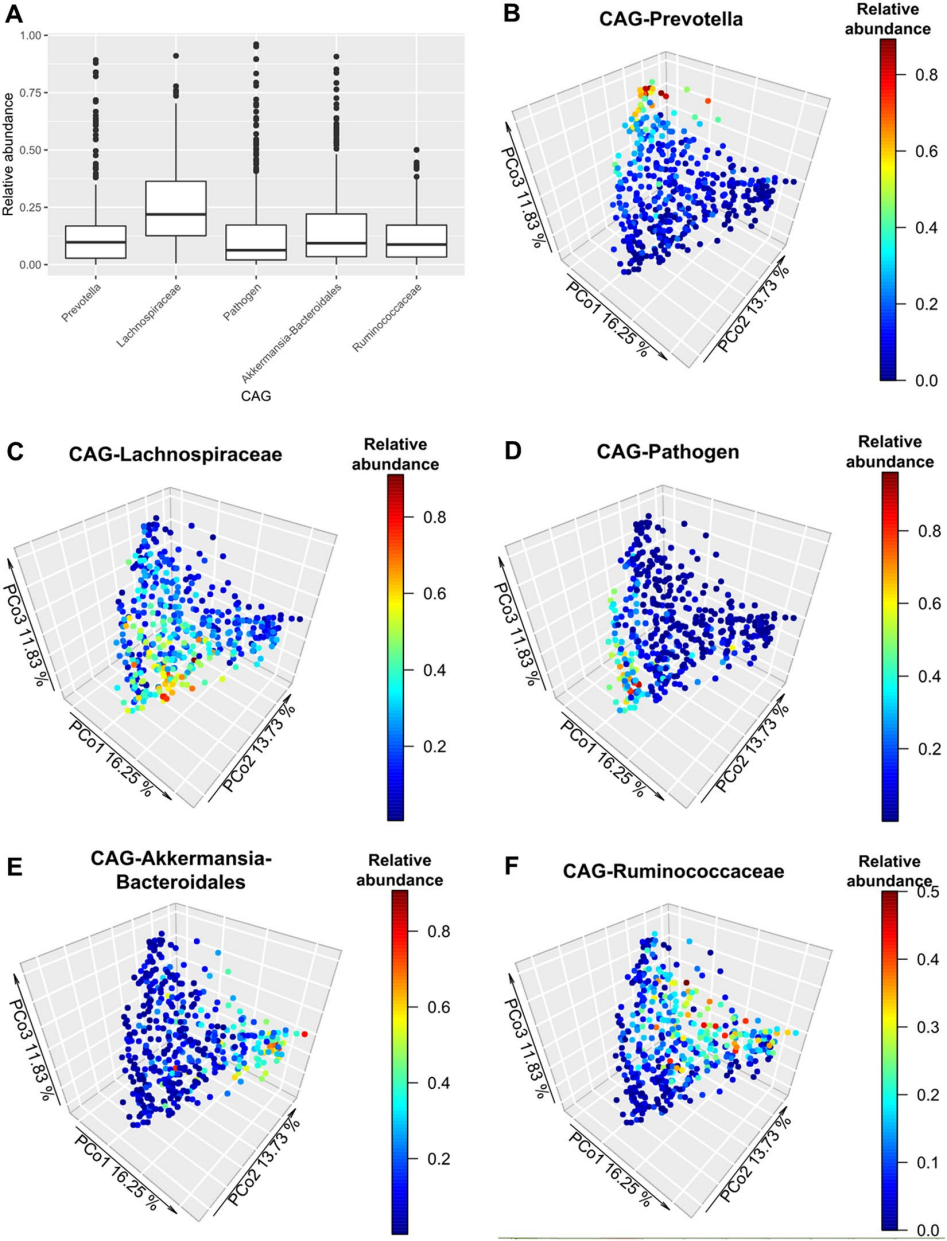
Abundance distribution of co-abundance groups (CAGs). **(A)** Distribution of the relative abundance of each CAG in the studied population (n = 441). **(B–F)** Principal coordinate analysis (PCoA) based on weighted UniFrac distances describing the enterogradient of the studied population (n = 441). The different panels show the same cloud point colored by the relative abundance of each co-abundance group (CAG). **(B)** Prevotella-CAG, **(C)** Lachnospiraceae-CAG, **(D)** Pathogen-CAG, **(E)** Akkermansia-Bacteroidales-CAG, **(F)** Ruminococcaceae-CAG. Percentages on the axes represent the proportion of the explained variation of each component of the PCoA. Note the change in the relative abundance scale among panels.

To manage this complexity, we clustered the above 100 most abundant OTUs into five co-abundance groups (CAGs)^21^ (Fig. S1). CAGs were defined by calculating Spearman’s correlation coefficients between all the aforementioned OTUs and by applying hierarchical clustering with Ward’s linkage^22^. OTUs with the highest median abundances served to name each CAG. The CAG clustering was confirmed by randomly partitioning the dataset (Mantel test with 10,000 permutations and 10,000 bootstrap iterations for the confidence intervals: r = 0.844; 95% CI [0.833, 0.855]; p = 0.0001) and by compositional network reconstruction using SparCC^23^. Note that CAGs represent sets of microorganisms exhibiting positive abundance correlations that thoroughly capture the continuous configuration of the gut microbiota (Fig. 3). Note also that CAGs are not a unique feature of our dataset; they have been shown in previous studies^21,22^ and confirmed by us in the meta-analysis of benchmark datasets mentioned above (Supplementary Results). The microbiota of most Colombians consisted of a variable combination of five CAGs (Fig. 3A), corroborating the continuous distribution in microbial composition.

The Prevotella-CAG comprised 9 OTUs that belong to the *Prevotella* genus and the Coriobacteriaceae family. The Lachnospiraceae-CAG included 32 OTUs belonging to the Lachnospiraceae family, such as *Roseburia*, *Blautia*, *Dorea* and *Coprococcus*, and high abundances of *Faecalibacterium* and *Gemmiger* (Ruminococcaceae). Most members of the Ruminococcaceae, however, clustered into the Ruminococcaceae-CAG, which included 21 OTUs, such as *Oscillospira* and *Ruminococcus*, but also the archaeon *Methanobrevibacter*. The Akkermansia-Bacteroidales-CAG comprised 26 OTUs, including *Akkermansia muciniphila*, *Bacteroides*, *Parabacteroides* and *Alistipes* (Bacteroidales). The Pathogen-CAG grouped 12 OTUs, including *Escherichia coli*, *Enterobacter hormaechei* and genera associated with the upper digestive tract, such as *Veillonella*, *Haemophilus*, *Gemella*, *Rothia*, *Burkholderia*, *Granulicatella* and *Streptococcus* (Table S5).

The members of these CAGs were not only phylogenetically but functionally related. The Lachnospiraceae-, Prevotella- and Ruminococcaceae-CAGs contained taxa associated with diets rich in fiber and complex carbohydrates^24^; members of the Akkermansia-Bacteroidales-CAG are involved in the degradation of mucins^25^; and members of the Pathogen-CAG are known opportunistic, potentially pathogenic bacteria known to contribute to various diseases^26^, including obesity and diabetes^27^, liver cirrhosis^28^, atherosclerotic cardiovascular disease^28,29^, colorectal cancer^30^ and anaerobic infections^31^. Interestingly, the species composition of the CAGs indicated that they are differentially associated with lifestyle: the Prevotella- and Ruminococcaceae-CAGs are enriched in species common in non-Western populations; the Lachnospiraceae-, Akkermansia-Bacteroidales- and Pathogen-CAGs in Western populations.

### CAGs associate with host health

A remarkable aspect of the five CAGs discussed above is that they do not distribute randomly along the enterogradient but tend to form foci where each of them is particularly abundant (Fig. 3B–F). To single out subjects with simple, distinctive microbiota arrangements in which one and only one of the five CAGs dominated the entire microbial community (in opposition to configurations in which the five CAGs had even abundances), we selected the subset of 114 individuals located on the highest extremes of the abundance distribution of each CAG (see Methods). This subset guaranteed the formation of non-overlapping groups of individuals located in the aforementioned foci. While reducing the dataset has the disadvantage of diminishing the representation of the cohort, it allowed assessing associations between broadly different gut microbiota configurations and host characteristics. The distinct compositional nature of the gut microbiota of this subset of individuals was confirmed by training a Random Forest model to classify subjects belonging to the foci based on OTU profile; this model showed 96.5% reclassification accuracy.

**Figure 4:**
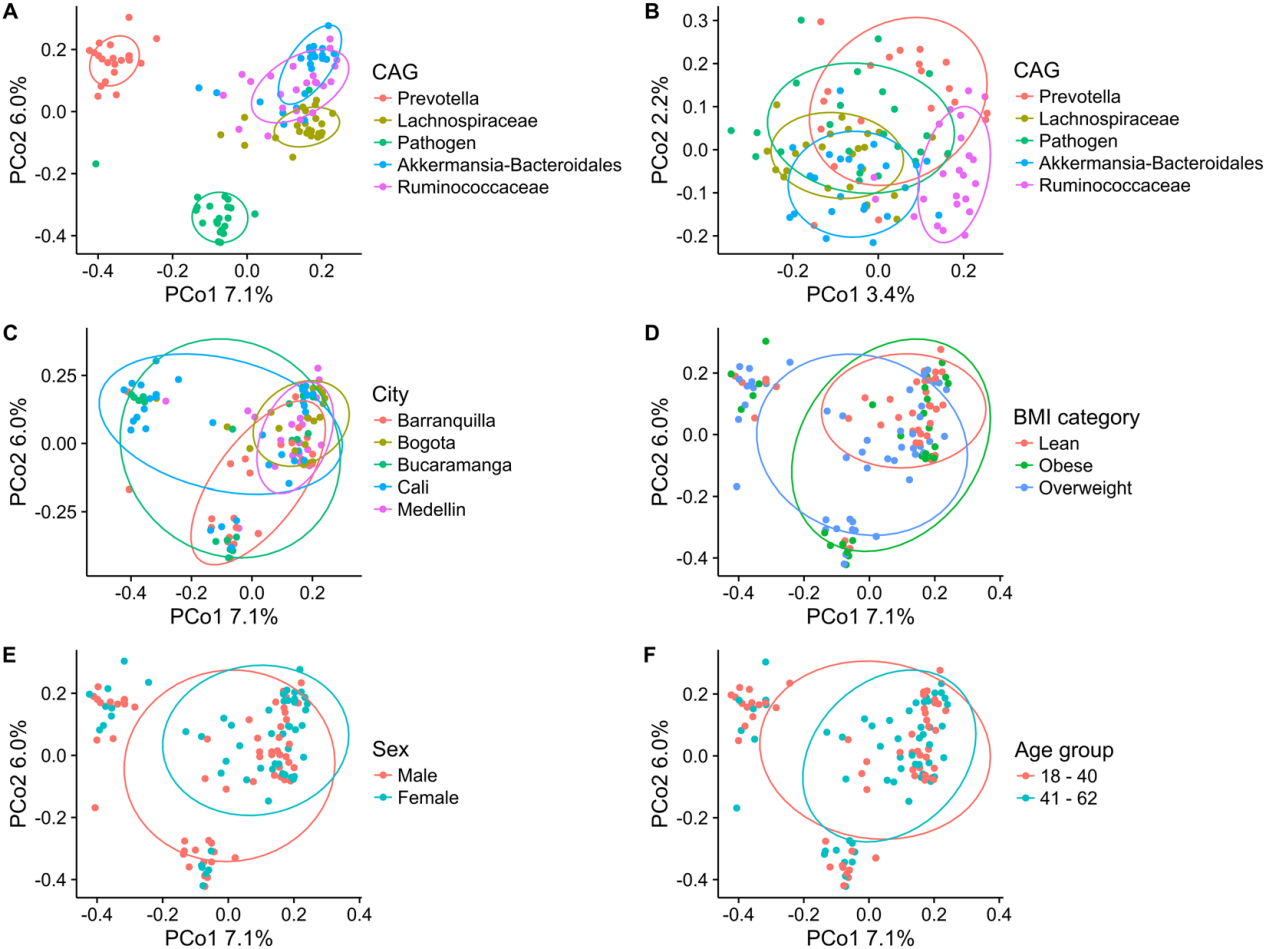
Composition of the total microbial community within the single-CAG dominated microbiota (n = 114) according to co-abundance groups (CAGs) (**A**,**B**), Colombian city of origin **(C)**, BMI **(D)**, sex **(E)** and age range **(F)**. All principal coordinate analyses (PCoA) were based on weighted UniFrac distances except in panel B (unweighted UniFrac). Ellipses encompass 75% of the variation. Percentages on the axes represent the proportion of explained variation of each component of the PCoA.

Ecological analyses in these 114 individuals showed that microbiota dominated by the Pathogen-, Akkermansia-Bacteroidales- and Prevotella-CAGs displayed the lowest α-diversities and contained some of the most abundant OTUs; the Lachnospiraceae- and Ruminococcaceae-CAGs had higher α-diversities (Fig. S2). The latter CAGs lacked a single dominant taxon, allowing more microbial groups to thrive with more homogenous abundances. In terms of β-diversity, the five CAGs explained a very high proportion of the variance in this subset of individuals when looking at the weighted UniFrac distance (PERMANOVA: R^2^ = 0.55, p = 0.01; Fig. 4A) and a lower but significant part with the unweighted UniFrac distance (R^2^ = 0.10, p = 0.01; Fig. 4B). This indicates that gut microbiota differences were caused primarily by alterations in the abundances of the microbes present rather than by changes in their membership.

Unlike common approaches in which individuals are first clinically classified (*e*.*g*., healthy *vs*. diseased) and then microbial differences are looked for, we agnostically distinguished individuals exclusively by their microbiota and then tested for differences in demographic, health-related and dietary variables. In this way, we determined whether the 114 individuals with single-CAG dominated microbiota were associated with variables indicative of the transition from traditional to Westernized lifestyles.

PERMANOVA tests indicated that the city where the samples originated was the most important factor in explaining the variance in the structure of the gut microbiota (participants were recruited in five Colombian cities; R^2^ = 0.113, p = 0.001; Fig. 4C), followed by the BMI (lean, overweight and obese; R^2^ = 0.034, p = 0.009; Fig. 4D) and the sex (R^2^ = 0.018, p = 0.038; Fig. 4E); no significant differences were observed by age range (18–40 and 41–62 years; R^2^ = 0.006, p = 0.69; Fig. 4F). Interestingly, anthropometric and health-related variables indicated that participants whose microbiota were dominated by the Ruminococcaceae- and the Akkermansia-Bacteroidales-CAGs had lower risks of obesity and cardiometabolic disease, whereas individuals whose microbiota were dominated by the Pathogen-CAG had increased risks of these diseases. Table 1 shows that there were significant differences associated to the microbiota composition according to BMI, waist circumference, blood pressure and adiponectin. Furthermore, individuals dominated by the Pathogen-CAG were characterized by a high proportion of diarrheic stools, suggestive of dysbiosis.

**Table 1:** General, anthropometric, health-related and dietary characteristics of the CAGs evaluated on the 114 individuals with single-CAG dominated microbiota.

	CAG					q-value
	Prevotella	Lachnospiraceae	Pathogen	Akkermansia-Bacteroidales	Ruminococcaceae	
n	22	23	23	23	23	
Age (years)	34.8 ± 11.7	39.2 ± 12.0	41.3 ± 12.6	41.7 ± 12.3	43.1 ± 9.2	0.142
Sex						
Male (%)	59.1	56.5	69.6	39.1	39.1	0.195
Female (%)	40.9	43.5	30.4	60.9	60.9	
City						
Barranquilla (%)	4.5	26.1	43.5	17.4	8.7	**0**.**004**
Bogota (%)	4.5	17.4	0.0	13.0	43.5	
Bucaramanga (%)	27.3	8.7	26.1	17.4	8.7	
Cali (%)	59.1	13.0	17.4	43.5	8.7	
Medellin (%)	4.5	34.8	13.0	8.7	30.4	
Anthropometric measures						
BMI (kg/m^2^)	28.7 ± 6.1	28.2 ± 4.4	29.5 ± 5.3	25.9 ± 4.6	25.8 ± 3.9	**0**.**055**
Body fat (%)	36.8 ± 7.1	37.5 ± 5.8	36.8 ± 5.1	36.2 ± 4.8	37.6 ± 5.5	0.568
Waist circumference (cm)	92.7 ± 13.9	94.9 ± 10.8	100.3 ± 15.5	86.5 ± 13.2	89.5 ± 12.2	**0**.**069**
Lipid profile						
Total cholesterol (mg/dL)	176.3 ± 31.5	183.3 ± 34.0	197.9 ± 47.1	190.7 ± 43.8	191.2 ± 39.1	0.334
HDL (mg/dL)	44.0 ± 9.1	42.5 ± 8.7	42.0 ± 13.6	47.8 ± 10.5	49.0 ± 12.1	0.318
LDL (mg/dL)	111.7 ± 27.4	114.7 ± 28.3	123.3 ± 42.7	113.7 ± 34.5	120.9 ± 35.1	0.521
Triglycerides (mg/dL)	118.8 ± 52.4	150.3 ± 83.7	180.8 ± 129.6	160.1 ± 163.7	120.8 ± 49.5	0.284
Adiponectin (µg/ml)	5.6 ± 1.5	5.8 ± 2.8	5.2 ± 2.7	7.8 ± 4.7	7.4 ± 4.1	**0**.**087**
Glucose metabolism						
Glucose (mmol/L)	86.3 ± 8.8	85.0 ± 7.1	91.0 ± 12.9	88.3 ± 22.0	89.7 ± 11.1	0.318
Insulin (µU/ml)	13.7 ± 8.1	14.2 ± 9.8	14.6 ± 6.9	11.0 ± 6.6	12.2 ± 8.1	0.318
Glycated hemoglobin (%)	5.5 ± 0.3	5.4 ± 0.4	5.5 ± 0.4	5.4 ± 0.5	5.6 ± 0.4	0.318
HOMA-IR	3.3 ± 2.2	2.5 ± 1.6	3.0 ± 1.6	2.8 ± 1.5	2.7 ± 2.0	0.323
Blood pressure (BP)						
Systolic BP (mm Hg)	124.6 ± 16.8	131.7 ± 22.4	133.9 ± 21.8	119.6 ± 14.3	115.2 ± 13.0	**0**.**014**
Diastolic BP (mm Hg)	76.5 ± 10.8	86.6 ± 12.8	86.7 ± 14.6	77.9 ± 11.4	72.7 ± 8.8	**0**.**002**
Inflammation						
hs-CRP (mg/L)	3.0 ± 2.9	4.4 ± 8.9	3.7 ± 4.0	2.4 ± 2.0	1.8 ± 1.3	0.318
Macronutrient consumption						
Total protein (%)	15.9 ± 1.4	15.2 ± 1.2	15.8 ± 1.9	15.8 ± 1.3	15.6 ± 1.4	0.390
Animal protein (%)	64.1 ± 5.4	61.0 ± 4.6	63.6 ± 6.0	63.4 ± 5.2	61.4 ± 6.0	0.277
Total fat (%)	29.9 ± 2.3	27.3 ± 2.1	28.5 ± 3.2	28.2 ± 2.3	29.2 ± 2.2	**0**.**033**
Saturated fat (%)	11.7 ± 1.3	11.0 ± 1.2	11.2 ± 2.0	11.1 ± 1.4	11.6 ± 1.4	0.284
Monounsaturated fat (%)	10.2 ± 0.9	9.4 ± 0.9	9.8 ± 1.1	9.8 ± 1.0	10.0 ± 0.9	**0**.**087**
Polyunsaturated fat (%)	5.9 ± 0.7	5.2 ± 0.8	5.4 ± 0.7	5.4 ± 0.9	5.5 ± 0.6	**0**.**087**
Carbohydrates (%)	54.0 ± 3.1	57.5 ± 2.1	55.6 ± 3.7	56.0 ± 2.5	55.2 ± 3.1	**0**.**014**
Dietary fiber (g)	19.1 ± 5.1	17.9 ± 4.3	17.8 ± 4.8	16.9 ± 4.1	16.4 ± 3.6	0.318
**Stool**						
Consistency						
Diarrheic (%)	9.1	0.0	13.0	4.3	0.0	**0**.**039**
Mushy (%)	4.5	21.7	30.4	13.0	8.7	
Normal (%)	81.8	73.9	34.8	52.2	73.9	
Hard (%)	4.5	4.3	21.7	30.4	17.4	
Fecal occult blood test						
Positive (%)	0.0	4.3	4.3	4.3	4.3	0.580
Negative (%)	100.0	95.7	95.7	95.7	95.7	
Medicament use						
Yes (%)	18.2	65.2	47.8	47.8	43.5	**0**.**069**
No (%)	81.8	34.8	52.2	52.2	56.5	

Data presented as the mean ± SD. BMI: body mass index, HDL: high density lipoprotein cholesterol, LDL: low density lipoprotein cholesterol, hs-CRP: high-sensitivity C-reactive protein, HOMA-IR: homeostatic model assessment–insulin resistance. q-values from ANOVA after false discovery rate correction.

While eloquent, the associations between microbiota and health detected in the subset of individuals with single-CAG dominated microbiota were limited by the reduction in the representation of the initial cohort (from 441 to 114 subjects). To assess whether these patterns hold when analyzing the complete cohort, we calculated correlations between the abundances of each CAG and health-related variables, and adjusted the p-values for multiple comparisons. We found similar results as those obtained in the reduced dataset, suggesting that the association between gut microbes and host health was not an artifact produced by the comparison of subsets of individuals with extreme configurations of the gut microbiota (Table 2).

**Table 2:** Correlations between α-diversity, health-related variables and CAG-abundance in the complete dataset (n = 441). Spearman’s rho and FDR-adjusted p-values (in parenthesis) are shown. Abbreviations as in Table 1.

	CAG				
	Prevotella	Lachnospiraceae	Pathogen	Akkermansia-Bacteroidales	Ruminococcaceae
Anthropometric measures					
BMI (kg/m^2^)	0.07 (0.31)	0.12 (0.05)	0.16 (0.002)	−0.16 (0.002)	−0.13 (0.03)
Body fat (%)	−0.02 (0.77)	0.07 (0.31)	0.00 (0.98)	−0.05 (0.50)	0.01 (0.90)
Waist circumference (cm)	0.10 (0.08)	0.09 (0.14)	0.16 (0.002)	−0.21 (<0.0001)	−0.12 (0.04)
Lipid profile					
Total cholesterol (mg/dL)	0.01 (0.95)	0.07 (0.28)	−0.03 (0.67)	0.01 (0.93)	0.00 (0.97)
HDL (mg/dL)	−0.12 (0.04)	0.02 (0.74)	−0.10 (0.09)	0.09 (0.15)	0.05 (0.43)
LDL (mg/dL)	0.04 (0.58)	0.06 (0.37)	−0.06 (0.34)	0.00 (0.97)	0.02 (0.74)
Triglycerides (mg/dL)	0.10 (0.10)	0.07 (0.25)	0.06 (0.37)	−0.07 (0.28)	−0.10 (0.09)
Adiponectin (µg/ml)	−0.15 (0.004)	0.02 (0.79)	−0.13 (0.02)	0.08 (0.20)	0.10 (0.11)
Glucose metabolism					
Glucose (mmol/L)	0.12 (0.04)	0.05 (0.47)	0.10 (0.08)	−0.11 (0.06)	−0.05 (0.48)
Insulin (µU/ml)	0.03 (0.71)	0.00 (0.95)	0.09 (0.16)	−0.08 (0.24)	−0.07 (0.25)
Glycated hemoglobin (%)	0.06 (0.37)	−0.08 (0.21)	−0.01 (0.88)	−0.02 (0.78)	0.07 (0.31)
HOMA-IR	0.08 (0.19)	−0.03 (0.67)	0.03 (0.65)	−0.05 (0.47)	−0.05 (0.42)
Blood pressure (BP)					
Systolic BP (mm Hg)	0.13 (0.02)	0.12 (0.04)	0.17 (0.002)	−0.18 (0.0008)	−0.22 (<0.0001)
Diastolic BP (mm Hg)	0.09 (0.12)	0.11 (0.06)	0.17 (0.001)	−0.16 (0.003)	−0.21 (<0.0001)
Inflammation					
hs-CRP (mg/L)	0.05 (0.47)	0.09 (0.16)	0.11 (0.05)	−0.08 (0.24)	−0.09 (0.12)
Macronutrient consumption					
Total protein (%)	−0.08 (0.21)	0.00 (0.95)	0.06 (0.37)	0.10 (0.11)	0.00 (0.97)
Animal protein (%)	−0.01 (0.93)	−0.07 (0.32)	0.09 (0.14)	0.03 (0.71)	−0.09 (0.13)
Total fat (%)	0.09 (0.16)	−0.03 (0.65)	0.03 (0.67)	0.01 (0.85)	−0.04 (0.62)
Saturated fat (%)	0.12 (0.05)	0.01 (0.88)	0.02 (0.84)	0.00 (0.95)	−0.06 (0.35)
Monounsaturated fat (%)	0.09 (0.18)	−0.05 (0.43)	−0.02 (0.80)	0.03 (0.70)	−0.03 (0.72)
Polyunsaturated fat (%)	0.07 (0.26)	−0.05 (0.51)	−0.01 (0.87)	0.01 (0.87)	−0.04 (0.64)
Carbohydrates (%)	−0.05 (0.42)	0.03 (0.65)	−0.05 (0.44)	−0.06 (0.35)	0.01 (0.88)
Dietary fiber (g)	0.06 (0.40)	0.04 (0.64)	−0.07 (0.25)	−0.04 (0.62)	0.04 (0.62)
Alpha diversity					
Species richness	0.02 (0.83)	−0.13 (0.03)	−0.29 (<0.0001)	0.13 (0.02)	0.71 (<0.0001)
Shannon index	0.11 (0.08)	0.18 (0.0008)	−0.23 (<0.0001)	−0.01 (0.88)	0.61 (<0.0001)
Pielou’s J	0.12 (0.04)	0.27 (<0.0001)	−0.18 (0.0006)	−0.05 (0.49)	0.52 (<0.0001)

### The functional potential of the gut microbiota reflects its species composition

**Figure 5:**
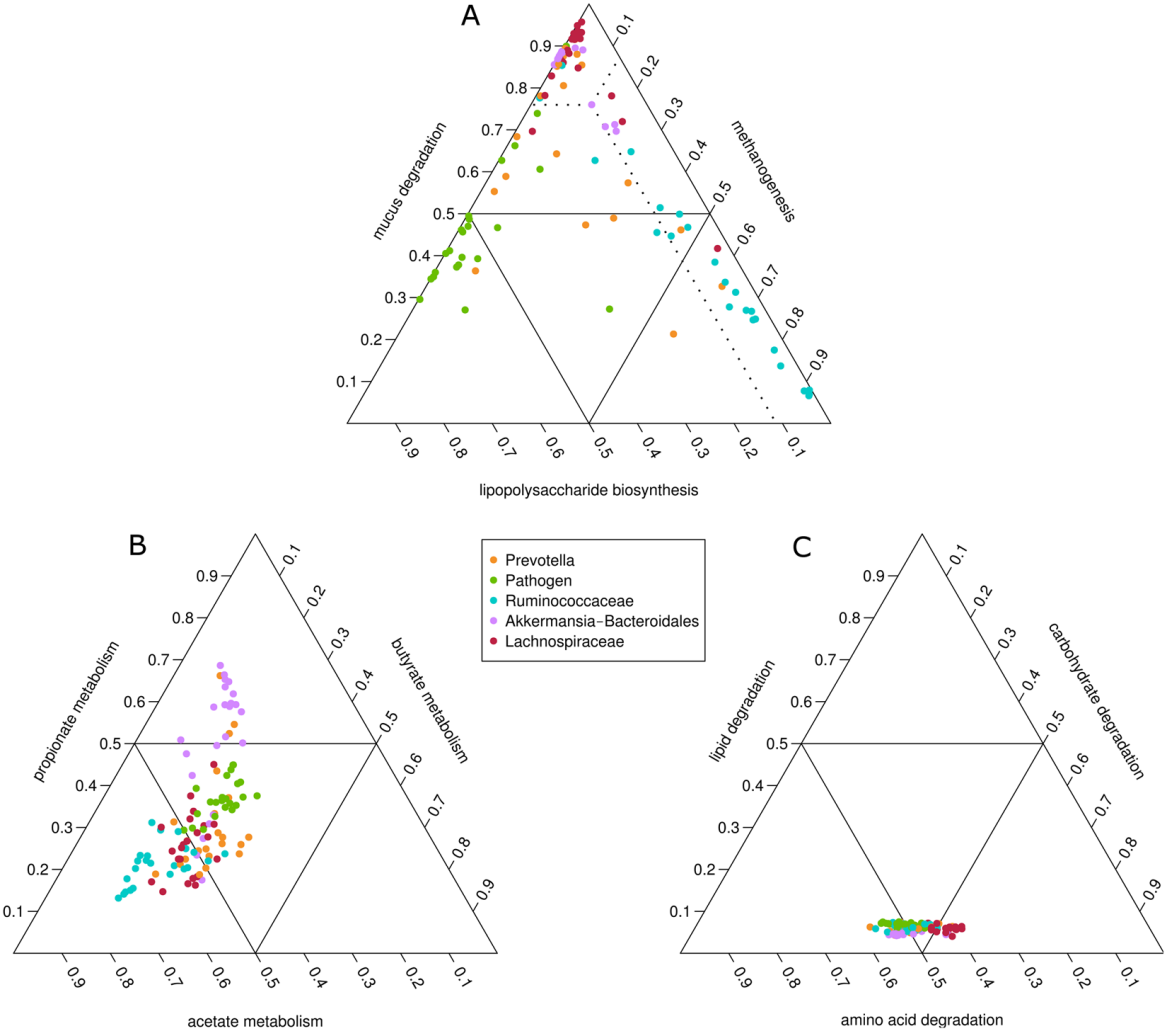
Predicted potential genomic investment of co-abundance groups (CAGs) within the subset of participants with single-CAG dominated microbiota (n = 114) in various relevant metabolic pathways. **(A)** Methane production, lipopolysaccharide biosynthesis and mucus degradation, **(B)** short-chain fatty acid production, **(C)** macronutrient degradation. Each point shows the relative abundance of the three metabolic processes depicted for a given individual. Dotted lines in panel **(A)** help in interpreting the figures by indicating how the values of a point project on the three axes. Note that for each point the values add up to one.

To better understand the functional potential of gut microbiota that might explain differences in disease risk, we inferred the metagenome of the 114 individuals with single-CAG dominated microbiota (Fig. S2D), summarized it into molecular function categories and determined the metabolic modules enriched in each microbial configuration (Table S6). These results were confirmed with the analysis of the complete dataset including 441 individuals (Table S7). The predicted genomic investment in various sets of biologically relevant modules was significantly different and clearly discriminant among CAGs, including pathways related to mucus degradation, methanogenesis and lipopolysaccharide (LPS) biosynthesis (Fig. 5A), as well as pathways related to the production of short-chain fatty acids (SCFAs) (Fig. 5B). We focused on these pathways since they have been largely shown to be relevant to host health. Mucin degradation has been associated with improved metabolic regulation, reduced obesity and type 2 diabetes^25^; LPS has been associated with metabolic endotoxemia, inflammation, insulin resistance, adiposity and hepatic fat^27^; methanogenesis seems to be key for ensuring complete fermentation of complex polysaccharides, leading to higher production and absorption of SCFAs^32^; and extensive evidence indicates that SCFAs are beneficial for the host cardiometabolic health^33^. In contrast, pathways related to central metabolism (*e*.*g*., glycolysis, pentose phosphate pathway, citric acid cycle) hardly differed among CAGs (Fig. 5C). Differences in functional potential reflect the compositions of the CAGs and strengthen the idea that these microbial consortia are composed of functionally related organisms. At the same time, this metagenomic inference proves that while extensive differences in the species composition of gut microbiota are accompanied by changes in particular metabolic pathways highly relevant for the host health, there is a strong functional redundancy in this community leading to conservation of a core metagenome (*e*.*g*., functions related with central metabolism)^3,34^.

## Discussion

We studied non-traditional, non-Western Colombians and showed that their gut microbiota forms a complex enterogradient, on which features of the microbiota of hunter-gatherers and citizens of industrialized countries can be identified. We detected several marker taxa distinctive in both Western and traditional microbiota, previously reported in studies and meta-analyses of benchmark metagenomic datasets. Based on the high *Prevotella* counts, Colombians possess a non-Western microbiota^17,35^; furthermore, the presence of *Treponema*, fiber-degrading bacteria enriched in rural communities and hunter-gatherers^7^, in addition to the high counts of Ruminococcaceae and *Methanobrevibacter*, strengthens this classification^17^. However, we also found high proportions of taxa that are common in Western populations but scarce in traditional communities, such as *Bacteroides*, *Bifidobacterium*, *Escherichia* and Lachnospiraceae, and a positive abundance of *Barnesiella*^17,35^. These commonalities suggest that the ancestral–Western transition is gradual and that broad lifestyle changes leave traces recognizable in the gut microbiota.

The complexity of this transitional microbiota can be comprehensively assessed through gradual changes in microbial consortia. We proved that our CAG clustering was robust to sampling stochasticity and clustering method. While the defined CAGs were composed of taxa recognized as common members of the human gut microbiota^3^, it is noticeable that the Prevotella- and Ruminococcaceae-CAGs were enriched in taxa proper of non-Western populations, whereas the Akkermansia-Bacteroidales- and Lachnospiraceae-CAGs enriched in taxa of Westerners. One of the most striking results of the CAG clustering was the detection of a consortium of co-occurring potentially pathogenic bacteria (Pathogen-CAG) that reached very high prevalence in some individuals (Fig. 3A); this CAG included Enterobacteriaceae and common members of the oral^3,36^ and stomach microbiota^37^. Patients with conditions such as liver cirrhosis^28^, atherosclerotic cardiovascular disease^28,29^, irritable bowel syndrome^38^, colorectal cancer^30^, anaerobic infections^31^ and diarrhea^39^ have increased counts of these microorganisms^26^. However, their presence^3^ and transcriptional activity^40^ have also been reported in the gut microbiota of Westernized community-dwelling individuals.

Unlike common approaches that examine the microbiota of individuals with contrasting clinical conditions, we exclusively distinguished members of our cohort by their gut microbiota and then tested whether microbial configurations were differentially associated with variables related to host health. This approach allowed the discovery of well-defined consortia of microorganisms associated with obesity and cardiometabolic risk, and metabolic pathways through which different microbiota could have an impact on health. It is worth stressing that microbial communities dominated by a single CAG represent upper tails of continuous distributions, not discrete configurations of the microbiota (*i*.*e*., they are not equivalent to enterotypes). This method uncovered clear-cut associations between health-related variables and simple microbial configurations in the context of continuous microbiota structures. Importantly, we showed that the conclusions drawn from this reduced dataset are extrapolatable to the complete dataset.

Our analyses revealed that the Pathogen- and Lachnospiraceae-CAGs were clearly associated with increased risk of cardiometabolic disease and obesity; individuals with high abundance of these CAGs had higher BMI, waist circumference and blood pressure, and lower adiponectin levels. Members of the Lachnospiraceae family have been shown to be associated with type 2 diabetes and obesity^41^, and *Enterobacter* and *Escherichia*, both gram-negative opportunistic pathogens grouping in the Pathogen-CAG, may be pivotal in obesity as the metabolic endotoxemia caused by LPS may induce inflammation, obesity and insulin resistance^27^. Our metagenomic inference indicated that the metabolic pathway involved in LPS biosynthesis was most abundant in the Pathogen-CAG.

In contrast, individuals with high abundance of Akkermansia-Bacteroidales- and Ruminococcaceae-CAGs had reduced risk of cardiometabolic disease and obesity, while having a metagenome enriched in pathways for mucin degradation and methane production, respectively. *Akkermansia muciniphila* has been consistently linked to improved metabolic health and leanness^25,42^; similarly, other members of the Akkermansia-Bacteroidales-CAG, such as *Alistipes* and *Bacteroides* isolated from lean mice, rapidly invade the microbiota of co-housed obese mice^43^. Members of the Ruminococcaceae-CAG, such as *Methanobrevibacter*, *Oscillospira* and *Dialister*, have also been associated with lower BMI^44–46^. The results from these two CAGs call into question the broader idea that low α-diversity and a Western microbiota are inherently associated with increased disease risk^47^: these two CAGs had contrasting α-diversities and contained dissimilar taxa related to the two ends of the Westernization spectrum. However, both CAGs were associated with host health.

The fact that distinct microbial consortia are associated with varying levels of disease risk illustrates that there are multiple ways through which the microbiota can affect health and disease. Strategies to promote the establishment and persistence of ‘healthy’ CAGs (*e*.*g*., Akkermansia-Bacteroidales- and Ruminococcaceae-CAGs) would be of great value in personalized nutrition and medicine, as they represent an intermediate state between the overwhelming complexity of modulating the whole microbial community and the reductionist approach of considering individual microbes; it could be a way to ameliorate conditions contributing to the burden of disease in Western societies.

## Methods

### Ethical approval

This study was conducted in accordance with the principles of the Declaration of Helsinki as revised in 2008 and had minimal risk according to the Colombian Ministry of Health (Resolution 8430 of 1993). All the participants were thoroughly informed about the study and procedures before signing consent forms. Participants were assured of anonymity and confidentiality. Written informed consent was obtained from all the participants before beginning the study. The Bioethics Committee of SIU—University of Antioquia reviewed the protocol and the consent forms and approved the procedures described here (approbation act 14-24-588 dated 28 May 2014).

### Study population

Between July and November 2014, we enrolled 441 men and women 18–62 years old, with BMI ≥ 18.5 kg/m^2^, living in the Colombian cities of Bogota, Medellin, Cali, Barranquilla and Bucaramanga, the country’s largest urban centers. All participants included in the study were insured by the health insurance provider EPS SURA. We excluded underweight participants (*i*.*e*., BMI < 18.5 kg/m^2^), pregnant women, individuals who had consumed antibiotics or antiparasitics in the three months prior to enrollment, and individuals diagnosed with any of the following diseases: Alzheimer’s disease, Parkinson disease or any other neurodegenerative disease; current or recent cancer (less than one year); and gastrointestinal diseases (Crohn’s disease, ulcerative colitis, short bowel syndrome, diverticulosis or celiac disease).

Participants were enrolled in roughly similar proportions by the city of origin, sex, age range (18–40 and 41–62 years) and BMI (lean, overweight and obese) (Table S2). In addition, individuals were randomly enrolled from two health-service-providing institutions (*Instituciones Prestadoras de Servicios*–IPS: residence proximity centers, clinics or hospitals where medical consultation services are rendered) of EPS SURA in four of the five cities (there was a unique IPS in Bucaramanga), with the aim of including intra-city variation in the study population.

### Blood biochemical parameters

For the measurement of clinical variables in blood serum, we collected fasting peripheral venous blood from all the participants and isolated the serum by centrifugation. Total cholesterol, high density lipoprotein (HDL) cholesterol, low density lipoprotein (LDL) cholesterol, triglycerides and fasting glucose were measured by colorimetric enzymatic assays (cobas 701; Roche, Mannheim, Germany); fasting insulin by a chemiluminescence immunoassay (cobas E411); glycated hemoglobin (HbA1C) by high-performance liquid chromatography (Premier Hb9210; Lab Care, England); adiponectin by the lanthanide chelate excite ultra assay (LANCE; Perkin Elmer, Waltham, MA), and high-sensitive C-reactive protein (hs-CRP) by a particle-enhanced immunoturbidimetric assay (cobas 502). Blood insulin was used to calculate the insulin resistance index using the homeostasis model assessment (HOMA-IR).

### Anthropometric evaluation and blood pressure

Weight, height, waist circumference and four skin folds (biceps, triceps, subscapular and ileocrestal) were measured with internationally recognized techniques after training and standardizing evaluators. Weight was measured with Cardinal Detecto DR400C digital scales (Webb City, MO) and height with Seca portable measuring rods (Hamburg, Germany). We calculated BMI as weight (kg)/height squared (m^2^) to classify participants as lean, overweight or obese. Waist circumference was measured with Mabis measuring tapes (Waukegan, IL) and skinfolds with Guide Slim adipometers (Plymouth, MI); skinfold measurements were used to calculate the fat percentage (the logarithm of the sum of the four folds allowed for a calculation of body density which was then used to estimate the body fat percentage using a validated equation^48^).

Blood pressure was measured using a Rossmax AF701f digital blood pressure monitor (Berneck, Switzerland); systolic and diastolic pressures were recorded in mm Hg. Each measure was evaluated twice, and the average of the two measures was reported.

### Diet assessment and medicament use

We carried out 24-hour dietary recall interviews to quantify calories and macronutrient intake in the habitual diet of participants. This method inquired about complete food and beverage descriptions, detailed preparation methods and portion sizes. Each participant was personally interviewed at least once by a trained member of the research team. Interviews were randomly distributed on different days of the week; ten percent of the participants were interviewed a second time on a different day of the week to assess intra-subject variability. Estimation of energy intake and macronutrients was obtained for each participant using the EVINDI 4.0 and PC-SIDE 1.0 software.

Pharmacological treatments were registered in specific questionnaires. By medicament use, we considered all drugs taken by participants on a regular basis during the three months prior to enrollment, to the exception of over-the-counter vitamin and mineral supplements, phytotherapeutics and contraceptives.

### Stool characterization, 16S rRNA PCR amplification and sequencing

Each participant collected a fecal sample in a hermetically sealed sterile receptacle provided by the research team. Samples were immediately refrigerated in household freezers and brought to an EPS SURA facility in each city within 12 hours, where they were stored in dry ice and sent to a central laboratory via next-day delivery. Stool consistency and the immunologic fecal occult blood test were performed on each sample.

Total microbial DNA was extracted using the QIAamp DNA Stool Mini Kit (Qiagen, Hilden, Germany) following the manufacturer’s instructions with a slight modification consisting of a bead-beating step with the lysis buffer (20 seconds at 15 Hz). After extraction, we quantified the DNA concentration using a Nanodrop spectrophotometer (Nyxor Biotech, Paris, France), and sent the DNA samples to the University of Michigan Medical School Host Microbiome Initiative (Ann Arbor, MI) for library construction and sequencing. The V4 hypervariable region of the 16S rRNA gene from each sample was amplified using primers F515 (5′-GTGCCAGCMGCCGCGGTAA-3′) and R806 (5′-GGACTACHVGGGTWTCTAAT-3′) and sequenced using the Illumina MiSeq sequencing platform with v2 chemistry and the dual-index sequencing strategy^49^.

### Sequence processing

We processed the 16S amplicon sequences using Mothur v.1.36 following its Illumina MiSeq standard operating procedure^49^. Briefly, we first extracted the sequence and quality score data from the paired fastq files and assembled the reads to form contigs. We eliminated sequences containing bases with a quality score below 20, sequences containing ambiguous bases, and sequences shorter than 275 bp. Next, we aligned the sequences using Silva reference alignment v.123^50^, which takes the secondary structure of the 16S rRNA into account, and removed sequences with a homopolymer run ≥8 nucleotides and sequences that did not overlap with the region of the alignment spanning the V4 hypervariable region. Then, we performed a preclustering step in which sequences with an identity ≥99% (*i*.*e*., sequences differing in 2 nucleotides or less) were merged. The chimeric sequences were detected and discarded by UCHIME^51^. After that, we assigned taxonomic classifications to the sequences using Greengenes^52^ 13_8_99 and removed sequences classified as mitochondria, eukaryota or unknown. Using the average neighbor algorithm, we generated operational taxonomic units (OTUs) delimited at 97% identity, which were taxonomically classified by consensus using Greengenes 13_8_99. A relaxed neighbor-joining tree with one representative sequence per OTU was finally obtained with Clearcut^53^ after calculating uncorrected pairwise distances between aligned reads.

### Quality control of microbial analysis

To examine and minimize the possible influence of reagent contamination, for each sequencing run, we included several technical controls, namely, a negative control (ultrapure water), a DNA extraction blank and a mock community (HM-782D, BEI Resources, Manassas, VA). In addition, we included the batch of the DNA extraction kit used with each sample as extra metadata and randomized the sequencing order of samples. To assess the reproducibility between sequencing runs, we included 5 replicate samples and determined the differences in the relative abundance of all OTUs (average difference between replicates ± SD: 0.01 ± 0.004%). Last, we calculated the Matthew’s Correlation Coefficient (MCC) to assess the stability and quality of the OTU assignments. MCC can be interpreted as representing the correlation between the observed and expected classifications, and it ranges from −1 to 1, where −1 represents total misclassification and 1 represents perfect classification. We obtained an MCC of 0.79, indicating high-quality OTU clustering.

### Analysis of Western and non-Western marker taxa

To verify the results found in the meta-analyses looking into the microbiota in the extremes of the Westernization spectrum, we performed an analysis using the curatedMetagenomicData^18^ package implemented in Bioconductor. This package provides uniformly processed and manually annotated human-microbiome profiles for thousands of subjects from benchmark studies. We downloaded gut microbiota taxonomic profiles of 16 publicly available studies and restricted this dataset to adult individuals with no report of disease or antibiotic consumption at the time of sampling. The final dataset comprised 1655 subjects from 16 countries, of which 1441 (87%) were considered Westernized and 214 (13%) non-Westernized (Table S3).

We obtained the relative abundances of taxa that can be used as markers of geographical origin and lifestyle in both the Colombian dataset and the 16 public datasets of curatedMetagenomicData. These included *Prevotella* and *Treponema* (markers of non-Western populations), and *Bacteroides*, *Bifidobacterium* and *Barnesiella* (markers of Western populations). Student’s t tests were implemented to test the null hypothesis that the mean abundance of each marker taxa was not significantly greater than zero; t tests were also employed to compare the abundances of marker taxa in the Colombian and public datasets.

### Evaluation of enterotypes and of an enterogradient

We determined whether the gut microbiota of Colombians clustered into enterotypes using the protocol proposed by Arumugam *et al*.^19^. (R code available at http://enterotype.embl.de/), incorporating the modifications suggested by Koren *et al*.^20^. Briefly, using the OTU-level abundance profile, we calculated the square root of the Jensen-Shannon divergence and the weighted UniFrac distance, computed with the GUniFrac package of R^54^, to obtain distance matrices which were then used to cluster samples with the partitioning around medoids (PAM) algorithm. Next, we calculated the average silhouette index (SI) for all possible clusters from 2 to 20, taking 0.50 and 0.75 as thresholds for moderate and strong clustering, respectively.

Since SI < 0.2 (*i*.*e*., low clustering) was seen in all cases, we next assessed changes in the gut microbiota of Colombians along an enterogradient. Analyses were restricted to weighted UniFrac distances, as this metric incorporates phylogenetic and abundance information of the sampled microorganisms into the comparison of communities. We carried out a principal coordinate analysis (PCoA) and evaluated the correlation of each OTU that had a median abundance ≥0.01% across all samples (100 OTUs), with the first three axes of PCoA, using Spearman’s rank correlations. Among these most-abundant OTUs, we selected those that moderately correlated (rho < −0.3 or rho > 0.3) with at least one of the first three axes of PCoA. These axes were selected because together they accounted for more than 40% of the total variance; in addition, this representation was convenient as the visualization of the three enterotypes originally reported by Arumugam *et al*.^19^ was evidenced in the first three axes of the PCoA analysis. P-values were FDR-adjusted for multiple comparisons using the qvalue package of R.

### Definition of co-abundance groups of microbes (CAGs)

To consider the network aspect of the gut microbiota and to detect robust compositional patterns, we defined CAGs of microbes, that is, OTUs that are found together more frequently and that reflect the underlying structure shaping the microbiota. CAGs were defined by calculating Spearman’s correlation coefficients between the 100 OTUs that had median abundances ≥0.01% across all samples and by applying hierarchical clustering with Ward’s linkage. OTUs with the highest median abundances served to name each CAG. This grouping was validated by randomly splitting the OTU table and computing two separate correlation matrices; the correlation between these matrices was obtained using the Mantel test as implemented in the ecodist package of R, with 10,000 permutations and 10,000 bootstrap iterations for the confidence intervals. In addition, we inferred correlation networks using SparCC^23^, an alternative method for computing correlations in compositional data. SparCC correlations were computed in 20 iterations of the dataset of the 100 most abundant OTUs, and the median value of each pairwise correlation was obtained.

### Characterization of single-CAG dominated microbiota

To identify the factors that were significantly associated with particular configurations of the gut microbiota, we selected a subset of individuals located on the extremes of the abundance distribution of each CAG, that is, participants that had a microbiota composed of a single CAG at an abundance ≥95^th^ percentile of that CAG distribution. Therefore, when analyzing individuals whose microbiota were dominated by a single CAG (in opposition to configurations in which the five CAGs had even abundances), we reduced the dataset from 441 to 114 individuals.

We trained the Random Forest machine-learning algorithm, as implemented in Mothur v.1.37, to reclassify the microbiota of these 114 individuals with extreme microbial configurations. For this, we used the *classify*.*rf* function with 1,000 trees, with the parameter controlling the aggressiveness of the reduced error pruning algorithm set to 0.9 and an error threshold of 0.4 to discard erroneous trees.

To characterize the microbial communities of the single-CAG dominated microbiota, α-diversity metrics were compared using the Shannon index, species richness, and Pielou’s J (evenness estimator), as implemented in the BiodiversityR R package. We tested for differences among CAGs using ANOVA and Tukey’s honest significance test for multiple comparisons. Next, we assessed differences in β-diversity estimates using the adonis function (analysis of variance using distance matrices) of the permutational multivariate analysis of variance (PERMANOVA) on the weighted and unweighted UniFrac matrices, as implemented in the Vegan package of R. In addition, to assess the effect of the Colombian city of origin (Bogota, Medellin, Cali, Barranquilla and Bucaramanga), sex (male, female), age range (18–40, 41–62 years), and BMI (lean, overweight, obese) on the overall microbial community of these 114 individuals, we compared β-diversity estimates among groups of participants using PERMANOVA. Next, we determined the biochemical, health-related and dietary profiles of these 114 individuals whose microbiota was dominated by a single CAG. We contrasted several parameters among these groups using one-way ANOVA on log-transformed data and chi-squared tests; p-values were FDR-adjusted using the qvalue package of R. To corroborate the above results, we performed a correlation analysis between CAG abundance, on one hand, and α-diversity and health-related variables, on the other hand, using all individuals of the studied cohort (441 individuals). Spearman’s correlations were obtained, and p-values were FDR-adjusted using the psych package of R.

### Metagenomic inference

The functional potential of the gut microbiota was inferred with the Tax4Fun R package^55^ using the SILVA database v.123 as a reference. In this way, we obtained a prediction of the relative abundance of each Kyoto Encyclopedia of Genes and Genomes (KEGG) ortholog (KO). KOs were subsequently collapsed into metabolic modules (*i*.*e*., sets of tightly related enzymatic functions that represent cellular processes with defined input and output metabolites)^56^ using GOmixer v.1.7.3 (http://www.raeslab.org/omixer/). Differences among groups of individuals for each metabolic module and correlations between module and CAG abundances were determined using Kruskal-Wallis tests and Spearman’s correlations, respectively; p-values were FDR-adjusted. Triplots of some biologically relevant functions were obtained with the dedicated tool implemented by GOmixer.

### Data Availability

Raw DNA reads were deposited at the SRA-NCBI under BioProject PRJNA417579. The R code to reproduce statistical analyses is available at https://github.com/jsescobar/westernization.

## Electronic supplementary material

Supplementary Materials
