# Supplementary material for: Gut microbiota is associated with obesity and cardiometabolic disease in a population in the midst of Westernization
Source: Sci Rep. 2018 Jul 27;8:11356. doi: 10.1038/s41598-018-29687-x (PMC6063892; doi:10.1038/s41598-018-29687-x)
Supplement: Supplementary file 1 — Supplementary Materials [file 41598_2018_29687_MOESM1_ESM.pdf]

Title: Gut microbiota is associated with obesity and cardiometabolic disease in a population in the midst of Westernization

Authors:
Jacobo de la Cuesta-Zuluaga[a,d], Vanessa Corrales-Agudelo[a], Eliana P. Velásquez-Mejía[a], Jenny A. Carmona[b,e], José M. Abad[c], and Juan S. Escobar[a]*

Affiliations:
[a] Vidarium—Nutrition, Health and Wellness Research Center, Grupo Empresarial Nutresa, Calle 8 sur 50-67, 050023 Medellin, Colombia. [b] Dinámica IPS—Especialista en Ayudas Diagnósticas, Calle 27 45-109, 050021 Medellin, Colombia. [c] EPS SURA, Calle 49A 63-55, 050034 Medellin, Colombia. [d] Current address: Max Planck Institute for Developmental Biology—Max-Planck-Ring 5, 72076 Tübingen, Germany. [e] Current address: SURA Colombia, Medellin, Colombia.

***Corresponding author:** Juan S. Escobar
Address: Vidarium—Nutrition, Health and Wellness Research Center, Grupo Empresarial Nutresa, Calle 8 sur 50-67, 050023 Medellin, Colombia.
Telephone number: (+57-4) 2856600 ext. 44508
Email: jsescobar@serviciosnutresa.com
Correspondence and material requests should be addressed to this author.

Supplementary Results

Prevotella-Bacteroides co-exclusion.

A pattern commonly observed in microbiome studies has been the co-exclusion of *Prevotella* and *Bacteroides*[1–5], which has been suggested to suffice for describing enterotypes[6]. We took advantage of the curatedMetagenomicData[7] package to analyze the breadth of this co-exclusion in 16 benchmark metagenomic studies. This meta-analysis confirmed the co-exclusion between these two taxa, with the co-exclusion being stronger in Western (Spearman's rho=-0.32, p=0.002) than in non-Western populations (rho=-0.21, p<0.001). We observed that the negative correlation between *Prevotella* and *Bacteroides* in the Colombian cohort was intermediate between Western and non-Western populations (Spearman's rho=-0.26, p<0.0001); this co-exclusion did not distinguish clear types of microbiota (Fig. SR1).

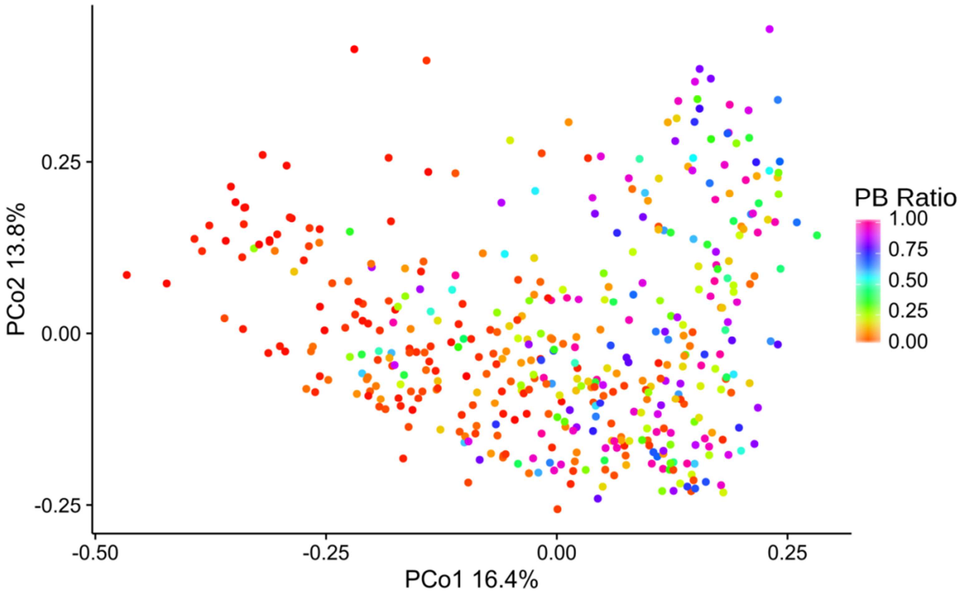

Fig. SR1. Principal coordinate analysis (PCoA) based on weighted UniFrac distances. Samples are colored by their *Prevotella-Bacteroides* ratio (PB ratio) using the relative abundances of all OTUs classified as *Prevotella* (174 OTUs) and *Bacteroides* (101 OTUs). The *Prevotella-Bacteroides* ratio was calculated as the relative abundances of *Prevotella*/(*Bacteroides* + *Prevotella*); red indicates no *Prevotella* and purple no *Bacteroides*. Percentages on the axes represent the proportion of the explained variation of each component of the PCoA.

Replicability of the CAG clustering.

We explored the replicability of the five detected CAGs underscored in the Colombian dataset using genus-level abundance in most of the benchmark metagenomic studies available in the curatedMetagenomicData[7] package (11 countries comprising 1600 individuals; we excluded datasets from Austria, Germany, Luxembourg, Peru and Tanzania since they considered few individuals for robust CAG inference); note that OTU abundance was unavailable in these datasets.

For each dataset, we applied the methodology employed for CAG definition in the Colombian dataset (see Methods in the main text), and compared the species composition of each CAG with those underscored in the Colombian cohort. In this way, we counted the number of times taxa clustered with "expected" microbes and the number of times there were "unexpected" associations. As an example, consider stool data from the Human Microbiome Project. In this dataset, we detected a CAG containing *Bifidobacterium*, *Collinsella*, *Coprococcus*, *Dorea*, *Faecalibacterium*, *Ruminococcus* and *Streptococcus*. All these microbes, except *Streptococcus*, clustered within the Lachnospiraceae-CAG in our Colombian cohort; *Streptococcus* was expected to cluster within the Pathogen-CAG. We counted the first six cases as checked (1 point was given to each taxon) while *Streptococcus* counted as unchecked (0 points). The replicability of a given CAG was calculated as the sum across datasets of all checked cases for the taxa "expected" to cluster in that CAG over the sum of checked and unchecked cases.

This analysis indicated that most datasets formed well-defined CAGs, some of which overlapped with the five CAGs uncovered in the Colombian cohort. In particular, the Akkermansia-Bacteroidales-, Pathogen- and Lachnospiraceae-CAGs were 70-80% replicable across datasets, whereas the Prevotella- and Ruminococcaceae-CAGs were less common (Table SR1). This is not surprising given that important taxa aggregating within the latter CAGs are enriched in non-Western populations (*e.g.*, *Prevotella*, Ruminococcaceae) while the datasets for comparison originated mostly from Westernized populations, where these taxa are rarer. Even though co-abundance patterns are fundamentally dataset-dependent, several of the CAGs that we underscored in the Colombian cohort were partly replicable and might represent general ecological associations in the human gut microbiota.

Table SR1. Replicability of the “Colombian” CAGs in publicly available datasets (see Methods in the main text).

CAG	Taxa “expected” to cluster within the CAG	Taxa found at least once in public datasets	Checked*	Checked + unchecked	Replicability of CAGs
Prevotella	6	4	7	16	43.75%
Akkermansia-Bacteroidales	17	14	75	94	79.79%
Ruminococcaceae	11	2	1	4	25.00%
Lachnospiraceae	18	9	58	84	69.05%
Pathogen	10	7	27	38	71.05%

*Number of times the taxa found in at least one public dataset clustered in the “expected” CAG across all datasets.

Supplementary References

1. Gorvitovskaia, A., Holmes, S. P. & Huse, S. M. Interpreting Prevotella and Bacteroides as biomarkers of diet and lifestyle. *Microbiome* **4,** 15 (2016).
2. Roager, H. M., Licht, T. R., Poulsen, S. K., Larsen, T. M. & Bahl, M. I. Microbial Enterotypes, Inferred by the Prevotella-to-Bacteroides Ratio, Remained Stable during a 6-Month Randomized Controlled Diet Intervention with the New Nordic Diet. *Appl. Environ. Microbiol.* **80,** 1142–1149 (2014).
3. Falony, G. *et al.* Population-level analysis of gut microbiome variation. *Science (80-.).* **352,** 560–564 (2016).
4. Koren, O. *et al.* A Guide to Enterotypes across the Human Body: Meta-Analysis of Microbial Community Structures in Human Microbiome Datasets. *PLoS Comput. Biol.* **9,** e1002863 (2013).
5. Dugas, L. R., Fuller, M., Gilbert, J. & Layden, B. T. The obese gut microbiome across the epidemiologic transition. *Emerg. Themes Epidemiol.* **13,** 2 (2016).
6. Arumugam, M. *et al.* Enterotypes of the human gut microbiome. *Nature* **473,** 174–180 (2011).
7. Pasolli, E. *et al.* Accessible, curated metagenomic data through ExperimentHub. *Nature Methods* **14,** 1023–1024 (2017).

Fig. S1. CAG-defining correlation heatmap. Dendrograms obtained by hierarchical Ward-linkage clustering based on Spearman's correlation coefficients of the relative abundances of the 100 OTUs that had median abundances ≥0.01%. Color bars on top and left of the heatmap show the defined CAGs.

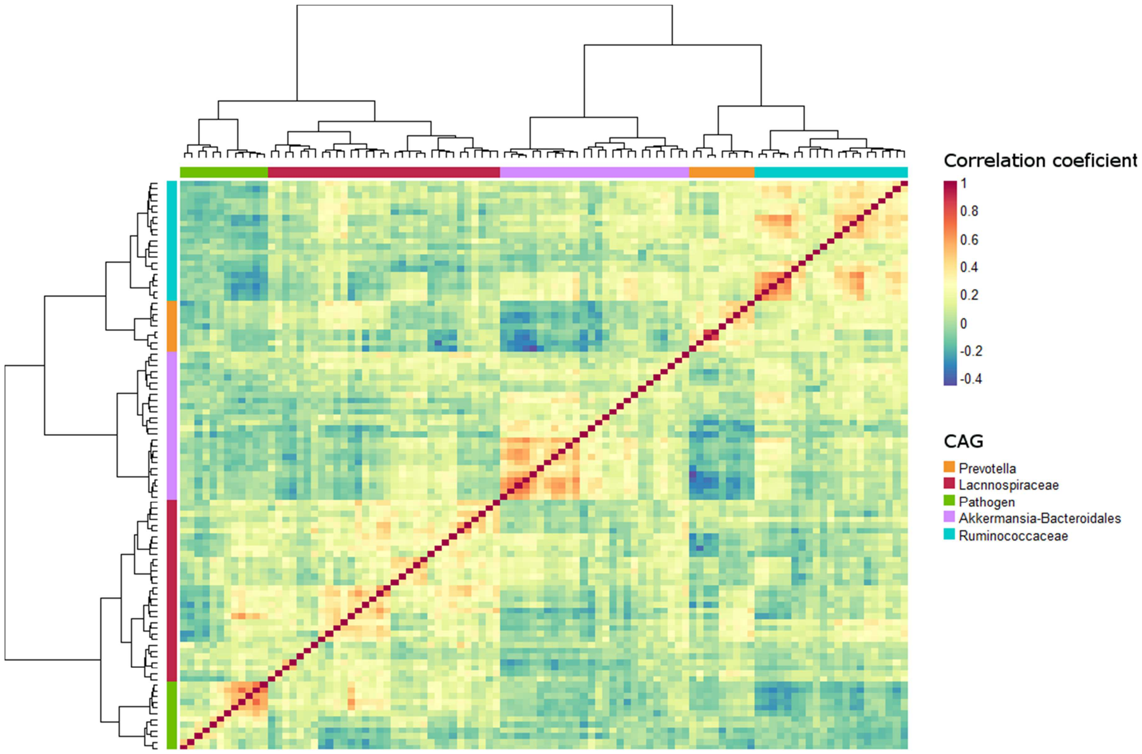

Fig. S2. Boxplot of α-diversity metrics of each co-abundance group (CAG) calculated in the subset of participants forming high-abundance poles (HAPs; n=114). (A) Species richness, (B) Shannon diversity index, (C) Pielou’s J, (D) KEGG ortholog (KO) richness. Notches represent the 95% confidence intervals of the median.

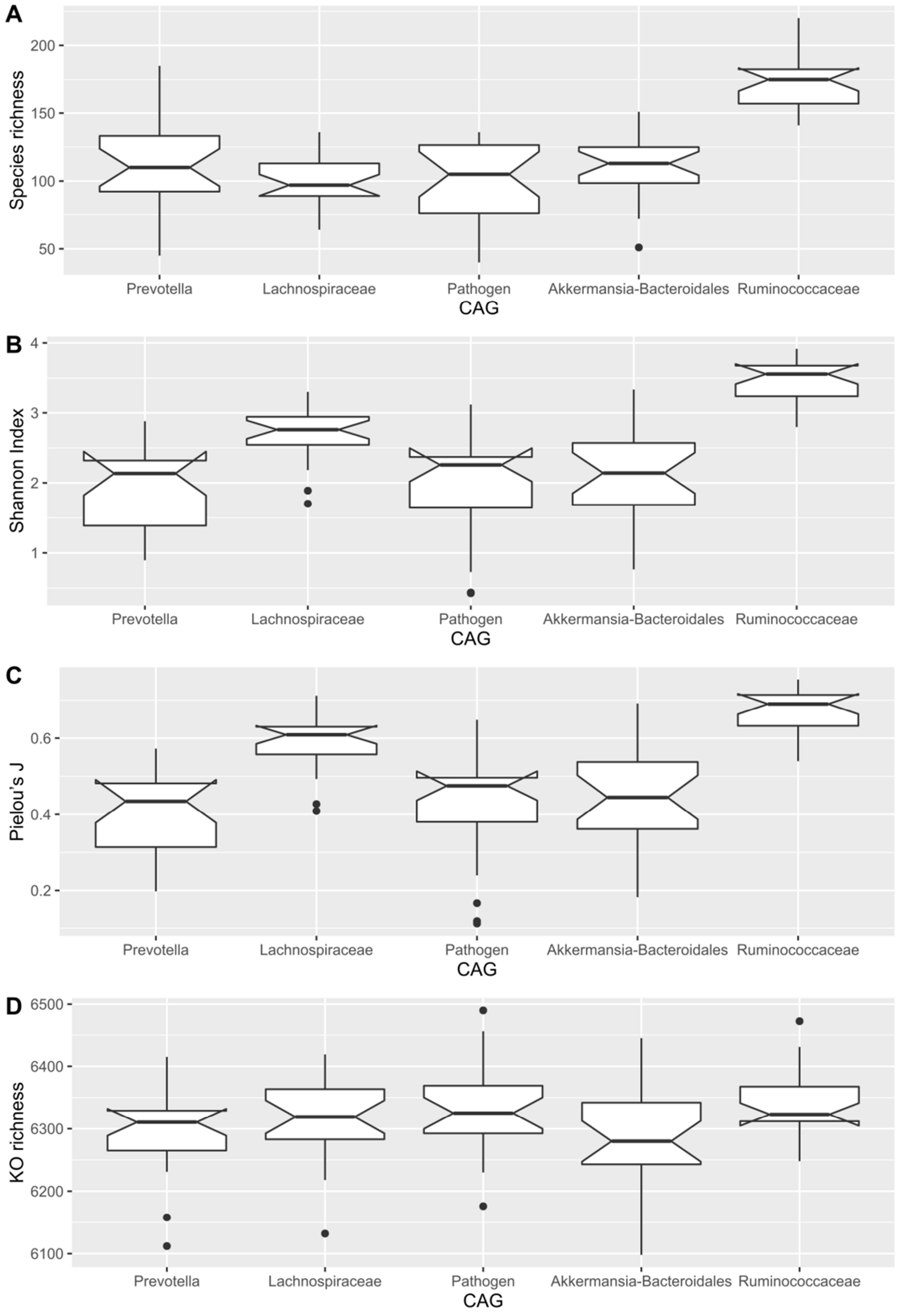

Table S1. Foods most commonly ingested by the participants of the study.

Food	Individuals (%)	Mean daily consumption (g)	Notes
Rice	77.8	150.2	
Vegetable oil	76.5	14.9	
Sugar	76.0	16.5	
Coffee	65.8	171.3	
Whole milk	62.3	228.1	
Tomatoes	58.4	57.5	
Potatoes	54.0	179	
Bread	48.6	70.9	
Eggs	47.7	79.4	
Beef	46.4	77.3	
Soda	44.2	336.9	
Arepa	43.1	96.4	Grilled patty of soaked, ground kernels of maize or maize flour
Cheese	41.0	52.9	
Vegetable fat	40.3	6.9	
Chicken	39.9	90.1	
Carrot	38.6	28.5	
Bulb onion	36.8	29.2	
Plantain	34.9	114.9	
Panela	30.3	46.3	Unrefined whole cane sugar
Cookies	30.3	33.1	
Lime	29.2	45.4	
Lunch meat	26.1	44.9	
Fish oil	24.4	8.8	
Tea	23.1	186.3	
Chocolate	21.4	9.9	
Lettuce	19.8	22.4	
Welsh onion	19.6	7.1	
Pea	18.3	32.8	
Green bean	15.7	31.1	
Pasta	15.7	133.6	
Banana	14.8	98	
Mango	14.8	138.7	
Papaya	14.4	98.6	
Bean	14.4	141.7	
Pork	14.4	81.3	
Coriander	13.3	0.9	
Garlic	12.9	2.1	
Tamarillo	12.4	77.7	*Solanum betaceum*
Blackberry	12.2	56.2	
Manioc	12.2	55.3	
Ketchup	11.8	12.9	
Snack foods	11.5	41.6	
Bell pepper	11.5	13.2	
Avocado	11.3	43.2	
Apple	10.7	141.3	
Cake	10.5	101.8	
Guava	10.2	115.3	
Orange	10.0	257.7	
Lentil	9.4	189.5	
Corn	9.2	38	
Lulo	9.2	91.3	*Solanum quitoense*
Pineapple	9.2	110.2	
Chorizo	8.7	33.2	
Breakfast cereal	8.3	30.2	
Oatmeal	8.1	66.6	
Toast	8.1	32.9	
Fish	7.8	141.6	
Cabbage	7.8	24.5	
Sugar candy	7.2	17.5	
Tangerine	7.2	252.4	
Empanada	7.0	149.6	Stuffed and fried pastry made of maize meal
Vinaigrette	6.5	4.5	
Animal fat	6.5	9	
Powdered milk	6.5	15.1	

Food	Individuals (%)	Mean daily consumption (g)	Notes
Goat cheese	6.3	48	
Organ meats	6.3	95	
Cucumber	6.1	51.3	
Chocolate bar	6.1	17.3	
Pastry	6.1	119.5	
Soft ice cream	5.9	89.8	
Bocadillo	5.7	27.7	Guava jelly with *panela*
Yogurt	5.4	227.2	
Canned tuna	5.4	115.2	
Passion fruit	5.2	103.1	
Bouillon cube	5.2	2.3	
Milk cream	5.0	10.7	
Peanuts	5.0	30.6	
Celery	4.8	19.7	
Strawberries	4.8	63.8	
Mustard	4.8	5.6	
Powdered drink mix	4.6	131	
Marmalade	4.6	12.4	
Squash	4.6	62.3	
Chewing gum	4.4	3.3	
Grapes	4.1	62.2	
Chili pepper	4.1	4	
Vinegar	3.9	2.1	
Buñuelo	3.9	75.3	Fried yeast dough with curd cheese
Pear	3.5	197.3	

For each food, the proportion of individuals who reported having eaten it in the last 24 hours and the mean intake in grams is given. Macronutrient intake in the studied population, expressed as the percentage of calories contributed by total carbohydrates, protein and total fat, was (mean ± SD) 55.4 ± 3.0%, 15.7 ± 1.4% and 28.7 ± 2.5%, respectively. Fiber intake was 17.7 ± 5.1 g.

Table S2. Proportions of enrolled participants according to the ciy of origin, sex, age range and BMI.

City of origin	N (%)	Sex (%male | %female)	Age range (%18-40 yrs | %41-62 yrs)	BMI (%Ln | %Ow | %Ob)
Barranquilla	89 (20%)	50 | 50	43 | 57	26 | 39 | 35
Bogota	85 (19%)	40 | 60	49 | 51	31 | 42 | 27
Bucaramanga	79 (18%)	52 | 48	62 | 38	38 | 39 | 23
Cali	89 (20%)	43 | 57	43 | 57	31 | 37 | 31
Medellin	99 (22%)	52 | 48	40 | 60	31 | 36 | 32
**Total**	**441 (100%)**	**48 | 52**	**47 | 53**	**31 | 39 | 30**

Ln=lean; Ow=overweight; Ob=obese

Table S3. Marker taxa associated with Westernization.

			Marker taxa				
**Country**	**Subjects**	**Lifestyle**	***Prevotella***	***Treponema***	***Bacteroides***	***Bifidobacterium***	***Barnesiella***
AUT	6	Western	0.1 ± 0.2	0 ± 0	4.9 ± 4.9	12.1 ± 5.2	0.5 ± 0 .5
CAN	36	Western	3.3 ± 13.1	0 ± 0	36 ± 18.3	1.3 ± 2.4	1.7 ± 2.5
CHN	278	Western	11.2 ± 21	0 ± 0	36 ± 21.3	1.2 ± 3	0.5 ± 1
DEU	5	Western	1.7 ± 3.8	0 ± 0	17.2 ± 10.2	7.5 ± 7.5	0.8 ± 0.6
DNK	163	Western	8 ± 16.4	0 ± 0	21.6 ± 14.6	1.1 ± 1.4	2.2 ± 2.7
ESP	63	Western	4.6 ± 12.7	0 ± 0	24 ± 13.8	3.1 ± 3.9	1.7 ± 1.5
FJI	112	Non-Western	31.5 ± 16.7	2.2 ± 9.1	1.7 ± 3.3	3.4 ± 6	0.1 ± 0.4
FRA	41	Western	3.1 ± 9.7	0 ± 0	15.1 ± 11.1	4.6 ± 5.5	0.8 ± 1.1
GBR	178	Western	1.3 ± 5.1	0 ± 0	21.6 ± 15.2	5.7 ± 7.7	2.5 ± 2.7
ITA	27	Western	5.1 ± 13.3	0 ± 0	11.7 ± 14.1	17.9 ± 25.1	0.5 ± 1.1
LUX	7	Western	0 ± 0	0 ± 0	19.6 ± 8.9	1.2 ± 1.1	3.2 ± 2.2
MNG	45	Western	17.1 ± 16.6	0 ± 0	5.2 ± 8.6	18.1 ± 17.4	0.2 ± 0.7
MNG	65	Non-Western	16 ± 14.3	0 ± 0	5.1 ± 10.1	17.8 ± 15.5	0.2 ± 0.4
NLD	393	Western	3.7 ± 7.7	0 ± 0.7	8.9 ± 7.8	15 ± 12.2	0.8 ± 1.1
PER	16	Non-Western	5.2 ± 4	8.8 ± 19.2	0.2 ± 0.3	2.9 ± 7.1	0 ± 0
TZA	21	Non-Western	27.6 ± 19.4	3.1 ± 3.5	0.1 ± 0.2	0 ± 0	0 ± 0
USA	199	Western	3.9 ± 11.6	0 ± 0	39.7 ± 24.6	1.1 ± 3.2	1.6 ± 3.1
		**Western**	**4.85 ± 4.80**	**0 ± 0**	**20.12 ± 11.52**	**6.92 ± 6.61 (5.98 ± 5.94)***	**1.31 ± 0.92**
		**Non-Western**	**20.08 ± 11.90**	**3.52 ± 3.75**	**1.78 ± 2.33**	**6.02 ± 7.99 (2.10 ± 1.84)***	**0.08 ± 0.10**
		**P-value****	**0.01**	**0.001**	**0.005**	**0.69 (0.35)***	**0.004**

Sample size, Westernization status and relative abundances of the marker taxa of the countries included in the analysis of publicly available datasets.

*The values in parentheses exclude the particular Mongolian populations.

**From Wilcoxon rank sum tests, testing the null hypothesis that the abundance of each marker taxa is not significantly different between Western and non-Western populations.

Table S4. OTUs correlated with the PCoA axes of weighted UniFrac distances.

		**Spearman's rho**		
**OTU**	**Taxonomy (k = kingdom; p = phylum; c = class; o = order; f = family; g = genus; s = species)**	**PCo1 (16.30%)**	**PCo2 (13.71%)**	**PCo3 (11.82%)**
Otu00001	k__Bacteria;p__Bacteroidetes;c__Bacteroidia;o__Bacteroidales;f__Prevotellaceae;g__Prevotella;s__copri;	**-0.56**	-0.13	**0.45**
Otu00002	k__Bacteria;p__Bacteroidetes;c__Bacteroidia;o__Bacteroidales;f__Prevotellaceae;g__Prevotella;s__copri;	**-0.65**	-0.15	**0.51**
Otu00003	k__Bacteria;p__Firmicutes;c__Clostridia;o__Clostridiales;f__Ruminococcaceae;g__Gemmiger;s__formicilis;	0.17	**0.51**	0.06
Otu00004	k__Bacteria;p__Proteobacteria;c__Gammaproteobacteria;o__Enterobacteriales;f__Enterobacteriaceae;g__Escherichia;s__coli;	**-0.35**	0.07	**-0.63**
Otu00006	k__Bacteria;p__Verrucomicrobia;c__Verrucomicrobiae;o__Verrucomicrobiales;f__Verrucomicrobiaceae;g__Akkermansia;s__muciniphila;	**0.48**	**-0.54**	-0.28
Otu00007	k__Bacteria;p__Actinobacteria;c__Actinobacteria;o__Bifidobacteriales;f__Bifidobacteriaceae;g__Bifidobacterium;s__adolescentis;	**0.33**	0.19	0.14
Otu00008	k__Bacteria;p__Firmicutes;c__Clostridia;o__Clostridiales;f__Ruminococcaceae;g__Faecalibacterium;s__prausnitzii;	**0.42**	**0.48**	0.07
Otu00009	k__Bacteria;p__Proteobacteria;c__Gammaproteobacteria;o__Enterobacteriales;f__Enterobacteriaceae;g__Enterobacter;s__hormaechei;	**-0.38**	0.23	**-0.38**
Otu00010	k__Bacteria;p__Firmicutes;c__Clostridia;o__Clostridiales;f__Clostridiaceae;g__Clostridium;s__celatum;	0.05	**0.30**	0.06
Otu00012	k__Bacteria;p__Firmicutes;c__Clostridia;o__Clostridiales;f__Ruminococcaceae;g__Oscillospira;unclassified;	0.16	-0.18	**0.35**
Otu00020	k__Bacteria;p__Firmicutes;c__Bacilli;o__Lactobacillales;f__Streptococcaceae;g__Streptococcus;unclassified;	0.18	**0.46**	-0.09
Otu00021	k__Bacteria;p__Firmicutes;c__Clostridia;o__Clostridiales;f__Ruminococcaceae;g__Oscillospira;unclassified;	-0.09	-0.08	**0.31**
Otu00022	k__Bacteria;p__Firmicutes;c__Bacilli;o__Lactobacillales;f__Streptococcaceae;g__Streptococcus;unclassified;	0.10	**0.35**	-0.14
Otu00030	k__Bacteria;p__Firmicutes;c__Clostridia;o__Clostridiales;f__Lachnospiraceae;g__Blautia;s__obeum;	**0.34**	**0.42**	0.10
Otu00032	k__Bacteria;p__Bacteroidetes;c__Bacteroidia;o__Bacteroidales;f__Prevotellaceae;g__Prevotella;s__stercorea;	**-0.38**	-0.02	0.19
Otu00033	k__Bacteria;p__Firmicutes;c__Clostridia;o__Clostridiales;f__Ruminococcaceae;g__Oscillospira;unclassified;	-0.02	-0.20	**0.32**
Otu00037	k__Bacteria;p__Firmicutes;c__Clostridia;o__Clostridiales;f__Lachnospiraceae;g__Blautia;unclassified;	0.00	**0.35**	0.05
Otu00041	k__Bacteria;p__Firmicutes;c__Clostridia;o__Clostridiales;f__Ruminococcaceae;g__Oscillospira;unclassified;	0.22	0.01	**0.32**
Otu00043	k__Bacteria;p__Firmicutes;c__Clostridia;o__Clostridiales;f__Ruminococcaceae;g__Oscillospira;unclassified;	-0.01	-0.17	**0.32**
Otu00046	k__Bacteria;p__Firmicutes;c__Clostridia;o__Clostridiales;f__Lachnospiraceae;g__Blautia;unclassified;	**0.30**	**0.37**	0.06
Otu00052	k__Bacteria;p__Actinobacteria;c__Coriobacteriia;o__Coriobacteriales;f__Coriobacteriaceae;unclassified;unclassified;	0.13	0.04	**0.30**
Otu00058	k__Bacteria;p__Actinobacteria;c__Actinobacteria;o__Actinomycetales;f__Actinomycetaceae;g__Actinomyces;unclassified;	**0.34**	**0.37**	0.04
Otu00060	k__Bacteria;p__Firmicutes;c__Clostridia;o__Clostridiales;f__Clostridiaceae;g__02d06;unclassified;	**0.36**	-0.06	0.20
Otu00083	k__Bacteria;p__Firmicutes;c__Clostridia;o__Clostridiales;f__Lachnospiraceae;g__Dorea;s__formicigenerans;	0.17	**0.46**	0.18
Otu00097	k__Bacteria;p__Firmicutes;c__Clostridia;o__Clostridiales;f__[Mogibacteriaceae];unclassified;unclassified;	**0.31**	0.29	0.17
Otu00108	k__Bacteria;p__Firmicutes;c__Clostridia;o__Clostridiales;f__Lachnospiraceae;g__Coprococcus;unclassified;	-0.10	-0.01	**0.34**
Otu00126	k__Bacteria;p__Firmicutes;c__Clostridia;o__Clostridiales;f__Lachnospiraceae;g__Clostridium;s__clostridioforme;	**0.37**	0.20	-0.02
Otu00197	k__Bacteria;p__Firmicutes;c__Bacilli;o__Gemellales;f__Gemellaceae;g__Gemella;unclassified;	0.12	**0.36**	-0.13
Otu00284	k__Bacteria;p__Firmicutes;c__Clostridia;o__Clostridiales;f__Lachnospiraceae;g__Coprococcus;unclassified;	0.22	**0.30**	0.24

Spearman’s correlation coefficients of the operational taxonomic units (OTUs) that had a median abundance ≥0.01% and that were significantly correlated (q-value<0.05) with one of the first three axes of the weighted UniFrac principal correspondence analysis (PCoA). Rho<-0.3 or rho>0.3, and q-value<0.05 are highlighted in bold. The percentages of explained variations are given for each PCoA axis.

Table S5. Clustering of the most abundant operational taxonomic units (OTUs) per co-abundance group (CAG).

CAG	95th percentile	OTU	Median relative abundance	Taxonomy (k = kingdom; p = phylum; c = class; o = order; f = family; g = genus; s = species)	SparCC-based CAG	Mean decrease accuracy
Prevotella	43.00%	Otu00001	0.003	k__Bacteria;p__Bacteroidetes;c__Bacteroidia;o__Bacteroidales;f__Prevotellaceae;g__Prevotella;s__copri;	Prevotella	0.698
		Otu00002	0.02472	k__Bacteria;p__Bacteroidetes;c__Bacteroidia;o__Bacteroidales;f__Prevotellaceae;g__Prevotella;s__copri;	Prevotella	1.457
		Otu00018	0.00101	k__Bacteria;p__Actinobacteria;c__Coriobacteriia;o__Coriobacteriales;f__Coriobacteriaceae;g__Adlercreutzia;unclassified;	Prevotella	0.052
		Otu00019	0.00128	k__Bacteria;p__Firmicutes;c__Erysipelotrichi;o__Erysipelotrichales;f__Erysipelotrichaceae;g__Catenibacterium;unclassified;	Prevotella	0.013
		Otu00032	0.0002	k__Bacteria;p__Bacteroidetes;c__Bacteroidia;o__Bacteroidales;f__Prevotellaceae;g__Prevotella;s__stercorea;	Prevotella	0.006
		Otu00034	0.00177	k__Bacteria;p__Firmicutes;c__Erysipelotrichi;o__Erysipelotrichales;f__Erysipelotrichaceae;g__[Eubacterium];s__biforme;	Prevotella	0.034
		Otu00047	0.00198	k__Bacteria;p__Actinobacteria;c__Coriobacteriia;o__Coriobacteriales;f__Coriobacteriaceae;g__Adlercreutzia;unclassified;	Prevotella	0.062
		Otu00052	0.00029	k__Bacteria;p__Actinobacteria;c__Coriobacteriia;o__Coriobacteriales;f__Coriobacteriaceae;unclassified;unclassified;	Prevotella	0.069
		Otu00075	0.00103	k__Bacteria;p__Actinobacteria;c__Coriobacteriia;o__Coriobacteriales;f__Coriobacteriaceae;g__Slackia;unclassified;	Prevotella	0.013
Lachnospiraceae	59.80%	Otu00003	0.01433	k__Bacteria;p__Firmicutes;c__Clostridia;o__Clostridiales;f__Ruminococcaceae;g__Gemmiger;s__formicilis;	Lachnospiraceae	0.113
		Otu00005	0.0102	k__Bacteria;p__Firmicutes;c__Clostridia;o__Clostridiales;f__Ruminococcaceae;g__Faecalibacterium;s__prausnitzii;	Pathogen	0.012
		Otu00007	0.00976	k__Bacteria;p__Actinobacteria;c__Actinobacteria;o__Bifidobacteriales;f__Bifidobacteriaceae;g__Bifidobacterium;s__adolescentis;	Lachnospiraceae	0.266
		Otu00008	0.01286	k__Bacteria;p__Firmicutes;c__Clostridia;o__Clostridiales;f__Ruminococcaceae;g__Faecalibacterium;s__prausnitzii;	Lachnospiraceae	0.352
		Otu00010	0.00383	k__Bacteria;p__Firmicutes;c__Clostridia;o__Clostridiales;f__Clostridiaceae;g__Clostridium;s__celatum;	Lachnospiraceae	0.043
		Otu00014	0.00808	k__Bacteria;p__Firmicutes;c__Clostridia;o__Clostridiales;f__Lachnospiraceae;g__Roseburia;s__faecis;	Pathogen	0.031
		Otu00016	0.00515	k__Bacteria;p__Firmicutes;c__Clostridia;o__Clostridiales;f__Ruminococcaceae;g__Cellulosibacter;s__alkalithermophilus;	Prevotella	0.038
		Otu00017	0.01072	k__Bacteria;p__Actinobacteria;c__Coriobacteriia;o__Coriobacteriales;f__Coriobacteriaceae;g__Collinsella;s__aerofaciens;	Lachnospiraceae	0.103
		Otu00024	0.00453	k__Bacteria;p__Firmicutes;c__Clostridia;o__Clostridiales;f__Lachnospiraceae;g__[Ruminococcus];s__gnavus;	Lachnospiraceae	0.022
		Otu00029	0.00296	k__Bacteria;p__Firmicutes;c__Clostridia;o__Clostridiales;f__Clostridiaceae;g__SMB53;unclassified;	Lachnospiraceae	0.022
		Otu00030	0.00182	k__Bacteria;p__Firmicutes;c__Clostridia;o__Clostridiales;f__Lachnospiraceae;g__Blautia;s__obeum;	Lachnospiraceae	0.136
		Otu00035	0.00149	k__Bacteria;p__Firmicutes;c__Clostridia;o__Clostridiales;f__Lachnospiraceae;g__Ruminococcus;s__lactaris;	Lachnospiraceae	0.004
		Otu00037	0.00264	k__Bacteria;p__Firmicutes;c__Clostridia;o__Clostridiales;f__Lachnospiraceae;g__Blautia;unclassified;	Lachnospiraceae	0.071
		Otu00040	0.00062	k__Bacteria;p__Firmicutes;c__Clostridia;o__Clostridiales;f__Ruminococcaceae;g__Butyricicoccus;s__pullicaecorum;	Pathogen	0.013
		Otu00045	0.00047	k__Bacteria;p__Cyanobacteria;c__Chloroplast;o__Streptophyta;unclassified;unclassified;unclassified;	Pathogen	0.019
		Otu00046	0.00117	k__Bacteria;p__Firmicutes;c__Clostridia;o__Clostridiales;f__Lachnospiraceae;g__Blautia;unclassified;	Lachnospiraceae	0.031
		Otu00048	0.00037	k__Bacteria;p__Firmicutes;c__Clostridia;o__Clostridiales;f__Lachnospiraceae;g__Clostridium;s__clostridioforme;	Pathogen	0
		Otu00057	0.00025	k__Bacteria;p__Firmicutes;c__Clostridia;o__Clostridiales;f__Lachnospiraceae;g__Coprococcus;unclassified;	Lachnospiraceae	0.012
		Otu00058	0.00049	k__Bacteria;p__Actinobacteria;c__Actinobacteria;o__Actinomycetales;f__Actinomycetaceae;g__Actinomyces;unclassified;	Lachnospiraceae	0.014
		Otu00059	0.00148	k__Bacteria;p__Firmicutes;c__Clostridia;o__Clostridiales;f__Lachnospiraceae;g__Blautia;unclassified;	Lachnospiraceae	0.032
		Otu00064	0.00149	k__Bacteria;p__Firmicutes;c__Clostridia;o__Clostridiales;f__Lachnospiraceae;g__Roseburia;s__inulinivorans;	Pathogen	0.007
		Otu00066	0.00088	k__Bacteria;p__Firmicutes;c__Clostridia;o__Clostridiales;f__Lachnospiraceae;g__Dorea;unclassified;	Lachnospiraceae	0.03
		Otu00068	0.00012	k__Bacteria;p__Firmicutes;c__Bacilli;o__Lactobacillales;f__Enterococcaceae;g__Enterococcus;s__casseliflavus;	Pathogen	0.007
		Otu00076	0.00039	k__Bacteria;p__Firmicutes;c__Clostridia;o__Clostridiales;f__Lachnospiraceae;g__Coprococcus;unclassified;	Pathogen	0.018
		Otu00083	0.00114	k__Bacteria;p__Firmicutes;c__Clostridia;o__Clostridiales;f__Lachnospiraceae;g__Dorea;s__formicigenerans;	Lachnospiraceae	0.117
		Otu00085	0.00157	k__Bacteria;p__Firmicutes;c__Clostridia;o__Clostridiales;f__Clostridiaceae;g__Clostridium;unclassified;	Lachnospiraceae	0.031
		Otu00097	0.00051	k__Bacteria;p__Firmicutes;c__Clostridia;o__Clostridiales;f__[Mogibacteriaceae];unclassified;unclassified;	Lachnospiraceae	0.044
		Otu00126	0.00072	k__Bacteria;p__Firmicutes;c__Clostridia;o__Clostridiales;f__Lachnospiraceae;g__Clostridium;s__clostridioforme;	Lachnospiraceae	0.057
		Otu00162	0.0001	k__Bacteria;p__Firmicutes;c__Clostridia;o__Clostridiales;f__Ruminococcaceae;g__Ruminococcus;unclassified;	Lachnospiraceae	0.018
		Otu00188	0.00012	k__Bacteria;p__Firmicutes;c__Clostridia;o__Clostridiales;f__Lachnospiraceae;g__Dorea;unclassified;	Lachnospiraceae	0.005
		Otu00201	0.00011	k__Bacteria;p__Firmicutes;c__Erysipelotrichi;o__Erysipelotrichales;f__Erysipelotrichaceae;g__Clostridium;s__ramosum;	Lachnospiraceae	0.087
		Otu00284	0.00013	k__Bacteria;p__Firmicutes;c__Clostridia;o__Clostridiales;f__Lachnospiraceae;g__Coprococcus;unclassified;	Lachnospiraceae	0.03
Pathogen	52.20%	Otu00004	0.02023	k__Bacteria;p__Proteobacteria;c__Gammaproteobacteria;o__Enterobacteriales;f__Enterobacteriaceae;g__Escherichia;s__coli;	Pathogen	0.514
		Otu00009	0.00218	k__Bacteria;p__Proteobacteria;c__Gammaproteobacteria;o__Enterobacteriales;f__Enterobacteriaceae;g__Enterobacter;s__hormaechei;	Pathogen	0.465
		Otu00020	0.00178	k__Bacteria;p__Firmicutes;c__Bacilli;o__Lactobacillales;f__Streptococcaceae;g__Streptococcus;unclassified;	Lachnospiraceae	0.14
		Otu00022	0.00129	k__Bacteria;p__Firmicutes;c__Bacilli;o__Lactobacillales;f__Streptococcaceae;g__Streptococcus;unclassified;	Lachnospiraceae	0.038
		Otu00044	0.0005	k__Bacteria;p__Firmicutes;c__Clostridia;o__Clostridiales;f__Veillonellaceae;g__Veillonella;s__dispar;	Pathogen	0.023
		Otu00071	0.00014	k__Bacteria;p__Bacteroidetes;c__Bacteroidia;o__Bacteroidales;f__Prevotellaceae;g__Prevotella;s__melaninogenica;	Pathogen	0.008
		Otu00073	0.00027	k__Bacteria;p__Proteobacteria;c__Gammaproteobacteria;o__Pasteurellales;f__Pasteurellaceae;g__Haemophilus;s__parainfluenzae;	Pathogen	0.011
		Otu00074	0.00017	k__Bacteria;p__Firmicutes;c__Bacilli;o__Lactobacillales;f__Streptococcaceae;g__Streptococcus;s__infantis;	Lachnospiraceae	0.026
		Otu00099	0.00016	k__Bacteria;p__Actinobacteria;c__Actinobacteria;o__Actinomycetales;f__Micrococcaceae;g__Rothia;s__mucilaginosa;	Pathogen	0.009
		Otu00113	0.00014	k__Bacteria;p__Proteobacteria;c__Betaproteobacteria;o__Burkholderiales;f__Burkholderiaceae;g__Burkholderia;unclassified;	Pathogen	0
		Otu00118	0.0001	k__Bacteria;p__Firmicutes;c__Bacilli;o__Lactobacillales;f__Carnobacteriaceae;g__Granulicatella;unclassified;	Lachnospiraceae	0.055
		Otu00197	0.00011	k__Bacteria;p__Firmicutes;c__Bacilli;o__Gemellales;f__Gemellaceae;g__Gemella;unclassified;	Lachnospiraceae	0.012

CAG	95th percentile	OTU	Median relative abundance	Taxonomy (k = kingdom; p = phylum; c = class; o = order; f = family; g = genus; s = species)	SparCC-based CAG	Mean decrease accuracy
Akkermansia-Bacteroidales	54.30%	Otu00006	0.007	k__Bacteria;p__Verrucomicrobia;c__Verrucomicrobiae;o__Verrucomicrobiales;f__Verrucomicrobiaceae;g__Akkermansia;s__muciniphila;	Akkermansia-Bacte	0.764
		Otu00013	0.0069	k__Bacteria;p__Firmicutes;c__Clostridia;o__Clostridiales;f__Ruminococcaceae;g__Ruminococcus;s__bromii;	Ruminococcaceae	0.02
		Otu00025	0.00159	k__Bacteria;p__Bacteroidetes;c__Bacteroidia;o__Bacteroidales;f__Bacteroidaceae;g__Bacteroides;unclassified;	Akkermansia-Bacte	0.041
		Otu00026	0.00158	k__Bacteria;p__Firmicutes;c__Clostridia;o__Clostridiales;f__Lachnospiraceae;g__Clostridium;s__hathewayi;	Akkermansia-Bacte	0.018
		Otu00027	0.00082	k__Bacteria;p__Bacteroidetes;c__Bacteroidia;o__Bacteroidales;f__Bacteroidaceae;g__Bacteroides;s__fragilis;	Akkermansia-Bacte	0.054
		Otu00049	0.00019	k__Bacteria;p__Bacteroidetes;c__Bacteroidia;o__Bacteroidales;f__Porphyromonadaceae;g__Parabacteroides;s__distasonis;	Akkermansia-Bacte	0.019
		Otu00050	0.00046	k__Bacteria;p__Bacteroidetes;c__Bacteroidia;o__Bacteroidales;f__Bacteroidaceae;g__Bacteroides;s__uniformis;	Akkermansia-Bacte	0.227
		Otu00056	0.00048	k__Bacteria;p__Firmicutes;c__Clostridia;o__Clostridiales;f__Ruminococcaceae;g__Subdoligranulum;s__variabile;	Akkermansia-Bacte	0.075
		Otu00062	0.00015	k__Bacteria;p__Bacteroidetes;c__Bacteroidia;o__Bacteroidales;f__[Paraprevotellaceae];g__Paraprevotella;unclassified;	Akkermansia-Bacte	0
		Otu00069	0.00012	k__Bacteria;p__Bacteroidetes;c__Bacteroidia;o__Bacteroidales;f__[Odoribacteraceae];g__Butyricimonas;unclassified;	Akkermansia-Bacte	0.026
		Otu00077	0.00016	k__Bacteria;p__Bacteroidetes;c__Bacteroidia;o__Bacteroidales;f__Bacteroidaceae;g__Bacteroides;s__caccae;	Akkermansia-Bacte	0.071
		Otu00080	0.00021	k__Bacteria;p__Bacteroidetes;c__Bacteroidia;o__Bacteroidales;f__Rikenellaceae;g__Alistipes;s__finegoldii;	Akkermansia-Bacte	0.008
		Otu00082	0.00015	k__Bacteria;p__Bacteroidetes;c__Bacteroidia;o__Bacteroidales;f__Rikenellaceae;g__Alistipes;s__putredinis;	Akkermansia-Bacte	0.052
		Otu00089	0.00015	k__Bacteria;p__Firmicutes;c__Clostridia;o__Clostridiales;f__Lachnospiraceae;g__Lachnospira;unclassified;	Ruminococcaceae	0.007
		Otu00092	0.00027	k__Bacteria;p__Bacteroidetes;c__Bacteroidia;o__Bacteroidales;f__Bacteroidaceae;g__Bacteroides;s__ovatus;	Akkermansia-Bacte	0.031
		Otu00095	0.00013	k__Bacteria;p__Firmicutes;c__Clostridia;o__Clostridiales;f__Lachnospiraceae;g__Dorea;unclassified;	Pathogen	0.008
		Otu00109	0.00014	k__Bacteria;p__Bacteroidetes;c__Bacteroidia;o__Bacteroidales;f__[Barnesiellaceae];unclassified;unclassified;	Akkermansia-Bacte	0.004
		Otu00110	0.00025	k__Bacteria;p__Firmicutes;c__Clostridia;o__Clostridiales;f__Lachnospiraceae;g__Coprococcus;unclassified;	Akkermansia-Bacte	0.003
		Otu00111	0.00029	k__Bacteria;p__Firmicutes;c__Clostridia;o__Clostridiales;f__Ruminococcaceae;g__Ruminococcus;s__albus;	Akkermansia-Bacte	0.056
		Otu00119	0.0001	k__Bacteria;p__Proteobacteria;c__Deltaproteobacteria;o__Desulfovibrionales;f__Desulfovibrionaceae;g__Desulfovibrio;unclassified;	Akkermansia-Bacte	0.028
		Otu00121	0.00029	k__Bacteria;p__Bacteroidetes;c__Bacteroidia;o__Bacteroidales;f__Porphyromonadaceae;g__Parabacteroides;unclassified;	Akkermansia-Bacte	0.035
		Otu00132	0.00021	k__Bacteria;p__Firmicutes;c__Clostridia;o__Clostridiales;f__Ruminococcaceae;unclassified;unclassified;	Ruminococcaceae	0.001
		Otu00175	0.0001	k__Bacteria;p__Firmicutes;c__Clostridia;o__Clostridiales;f__Ruminococcaceae;g__Ruminococcus;unclassified;	Akkermansia-Bacte	0.024
		Otu00177	0.00013	k__Bacteria;p__Proteobacteria;c__Deltaproteobacteria;o__Desulfovibrionales;f__Desulfovibrionaceae;g__Bilophila;unclassified;	Akkermansia-Bacte	0.025
		Otu00220	0.00015	k__Bacteria;p__Firmicutes;c__Clostridia;o__Clostridiales;f__Lachnospiraceae;g__Coprococcus;s__catus;	Pathogen	0.021
		Otu00228	0.0001	k__Bacteria;p__Bacteroidetes;c__Bacteroidia;o__Bacteroidales;f__[Odoribacteraceae];g__Odoribacter;unclassified;	Akkermansia-Bacte	0.012
Ruminococcaceae	32.20%	Otu00011	0.00122	k__Archaea;p__Euryarchaeota;c__Methanobacteria;o__Methanobacteriales;f__Methanobacteriaceae;g__Methanobrevibacter;unclassified;	Ruminococcaceae	0.407
		Otu00012	0.00996	k__Bacteria;p__Firmicutes;c__Clostridia;o__Clostridiales;f__Ruminococcaceae;g__Oscillospira;unclassified;	Ruminococcaceae	0.559
		Otu00015	0.00051	k__Bacteria;p__Firmicutes;c__Clostridia;o__Clostridiales;f__Ruminococcaceae;g__Ruminococcus;unclassified;	Ruminococcaceae	0.064
		Otu00021	0.00308	k__Bacteria;p__Firmicutes;c__Clostridia;o__Clostridiales;f__Ruminococcaceae;g__Oscillospira;unclassified;	Ruminococcaceae	0.025
		Otu00023	0.00017	k__Bacteria;p__Firmicutes;c__Clostridia;o__Clostridiales;f__Peptostreptococcaceae;g__[Clostridium];s__litorale;	Ruminococcaceae	0.033
		Otu00028	0.00054	k__Bacteria;p__Firmicutes;c__Clostridia;o__Clostridiales;f__Veillonellaceae;g__Dialister;unclassified;	Ruminococcaceae	0.058
		Otu00033	0.00066	k__Bacteria;p__Firmicutes;c__Clostridia;o__Clostridiales;f__Ruminococcaceae;g__Oscillospira;unclassified;	Ruminococcaceae	0.112
		Otu00041	0.00103	k__Bacteria;p__Firmicutes;c__Clostridia;o__Clostridiales;f__Ruminococcaceae;g__Oscillospira;unclassified;	Ruminococcaceae	0.168
		Otu00043	0.0009	k__Bacteria;p__Firmicutes;c__Clostridia;o__Clostridiales;f__Ruminococcaceae;g__Oscillospira;unclassified;	Ruminococcaceae	0.217
		Otu00060	0.00019	k__Bacteria;p__Firmicutes;c__Clostridia;o__Clostridiales;f__Clostridiaceae;g__02d06;unclassified;	Ruminococcaceae	0.1
		Otu00063	0.00175	k__Bacteria;p__Firmicutes;c__Clostridia;o__Clostridiales;f__Veillonellaceae;g__Propionispora;s__hippei;	Ruminococcaceae	0.044
		Otu00084	0.00032	k__Bacteria;p__Firmicutes;c__Clostridia;o__Clostridiales;f__Ruminococcaceae;unclassified;unclassified;	Ruminococcaceae	0.197
		Otu00090	0.00019	k__Bacteria;p__Firmicutes;c__Clostridia;o__Clostridiales;f__Ruminococcaceae;g__Ruminococcus;unclassified;	Ruminococcaceae	0.008
		Otu00102	0.00041	k__Bacteria;p__Firmicutes;c__Clostridia;o__Clostridiales;f__Ruminococcaceae;g__Oscillospira;unclassified;	Ruminococcaceae	0.04
		Otu00108	0.00014	k__Bacteria;p__Firmicutes;c__Clostridia;o__Clostridiales;f__Lachnospiraceae;g__Coprococcus;unclassified;	Ruminococcaceae	0.02
		Otu00112	0.00033	k__Bacteria;p__Firmicutes;c__Clostridia;o__Clostridiales;f__Ruminococcaceae;g__Oscillospira;unclassified;	Akkermansia-Bacte	0.009
		Otu00129	0.00031	k__Bacteria;p__Firmicutes;c__Clostridia;o__Clostridiales;f__Ruminococcaceae;g__Ruminococcus;unclassified;	Ruminococcaceae	0.081
		Otu00147	0.00033	k__Bacteria;p__Firmicutes;c__Clostridia;o__Clostridiales;f__Ruminococcaceae;g__Oscillospira;unclassified;	Ruminococcaceae	0.091
		Otu00148	0.00018	k__Bacteria;p__Firmicutes;c__Clostridia;o__Clostridiales;f__Ruminococcaceae;g__Ruminococcus;unclassified;	Ruminococcaceae	0.095
		Otu00151	0.0003	k__Bacteria;p__Firmicutes;c__Bacilli;o__Bacillales;f__Paenibacillaceae;g__Paenibacillus;s__ginsengarvi;	Ruminococcaceae	0.209
		Otu00325	0.00023	k__Bacteria;p__Firmicutes;c__Clostridia;o__Clostridiales;f__Lachnospiraceae;g__Clostridium;s__aerotolerans;	Ruminococcaceae	0.282

The 95th percentile of the distribution of each CAG is shown. In addition, for each OTU, the median relative abundance, the taxonomy, the alternative grouping proposed by the compositional network reconstruction analysis (SparCC) and the decreasing mean accuracy to discriminate CAGs by the Random Forest machine-learning algorithm are shown.

Table S6. Metabolic modules present in the gut microbiota of individuals with single-CAG dominated microbiota.

Metabolic module ID	Metabolic module name	Hierarchy level 1	Hierarchy level 2	Prevotella-CAG	Lachnospiraceae-CAG	Pathogen-CAG	Akkermansia-Bacteroidales-CAG	Ruminococcaceae-CAG	p-value (Kruskal-Wallis)	q-value (Benjamini-Hochberg)
MF0001	ethanol production (formate pathway)	alcohol metabolism	ethanol metabolism	0.001895	0.002039124	0.001441904	0.0012	0.00142	5.03E-09	6.86E-09
MF0003	acetylglucosamine degradation	amines and polyamines degradation	acetylglucosamine degradation	0.0003715	0.000389	0.000313	0.000514	0.000314	4.15E-12	9.23E-12
MF0004	putrescine degradation	amines and polyamines degradation	biogenic amine degradation	0.0000639	0.000119	0.00019	0.0000272	0.0000377	3.39E-12	7.94E-12
MF0005	acetylneuraminate and acetylmannosamine degradation	amines and polyamines degradation	sialic acid degradation	0.000131	0.0000773	0.000177	0.000205	0.0000893	4.52E-15	3.94E-14
MF0006	urea degradation	amines and polyamines degradation	urea degradation	0.0001735	0.000358	0.000238	0.0000981	0.000106	1.11E-08	1.43E-08
MF0007	phenylalanine degradation	amino acid degradation	aromatic amino acid degradation	0.0005235	0.000603	0.000399	0.000577	0.000611	3.18E-14	1.49E-13
MF0008	tyrosine degradation (hydroxyphenylacetaldehyde pathway)	amino acid degradation	aromatic amino acid degradation	0.000531	0.000606	0.000331	0.000634	0.000623	6.49E-14	2.57E-13
MF0009	tryptophan degradation	amino acid degradation	aromatic amino acid degradation	0.0002925	0.000129	0.000192	0.00111	0.000275	6.25E-13	1.70E-12
MF0010	tyrosine degradation (phenol pathway)	amino acid degradation	aromatic amino acid degradation	0.000017	0.00000889	0.0000169	0.00000374	0.0000061	4.45E-08	5.35E-08
MF0011	aspartate degradation (oxaloacetate pathway)	amino acid degradation	negatively charged amino acid degradation	0.000569	0.000522	0.000305	0.001096936	0.000887	5.95E-16	1.11E-14
MF0012	aspartate degradation (fumarate pathway)	amino acid degradation	negatively charged amino acid degradation	0.0003675	0.000492	0.000449	0.000135	0.000304	2.19E-07	2.47E-07
MF0013	glutamate degradation (crotonyl-CoA pathway)	amino acid degradation	negatively charged amino acid degradation	0.0002	0.000171	0.000124	0.000268	0.000199	4.11E-16	1.11E-14
MF0014	glutamate degradation (4-aminobutanoate pathway)	amino acid degradation	negatively charged amino acid degradation	0.000249	0.000293	0.000274	0.0011	0.000433	5.15E-08	6.14E-08
MF0015	glutamate degradation (methylaspartate pathway)	amino acid degradation	negatively charged amino acid degradation	0.00001275	0.00000265	0.0000354	0.00000175	0.0000036	3.71E-12	8.52E-12
MF0016	glycine degradation	amino acid degradation	nonpolar, aliphatic amino acid degradation	0.000469	0.0003	0.000617	0.00142	0.00047	7.71E-14	2.97E-13
MF0017	alanine degradation (racemase pathway)	amino acid degradation	nonpolar, aliphatic amino acid degradation	0.0005605	0.000582	0.000584	0.000812	0.000456	4.43E-14	1.87E-13
MF0018	proline degradation (glutamate pathway)	amino acid degradation	nonpolar, aliphatic amino acid degradation	0.000344	0.0000721	0.000891	0.00192	0.000246	3.12E-13	9.15E-13
MF0019	proline degradation (aminopentanoate pathway)	amino acid degradation	nonpolar, aliphatic amino acid degradation	0.000008125	0.00000317	0.00000461	0.000000888	0.00000193	1.69E-06	1.82E-06
MF0020	valine degradation	amino acid degradation	nonpolar, aliphatic amino acid degradation	0.0003165	0.000309	0.000363	0.000268	0.000299	1.81E-13	5.79E-13
MF0021	leucine degradation	amino acid degradation	nonpolar, aliphatic amino acid degradation	0.000205	0.000199	0.000194	0.000231	0.00023	1.40E-08	1.77E-08
MF0022	isoleucine degradation	amino acid degradation	nonpolar, aliphatic amino acid degradation	0.0003915	0.000348	0.000439	0.000301	0.000402	7.17E-13	1.92E-12
MF0023	methionine degradation (cysteine pathway)	amino acid degradation	nonpolar, aliphatic amino acid degradation	0.0009635	0.000891	0.000553	0.001064205	0.00109	2.38E-17	3.12E-15
MF0024	methionine degradation (mercaptan pathway)	amino acid degradation	nonpolar, aliphatic amino acid degradation	0.000137	0.000115	0.0000914	0.0000392	0.000119	1.08E-07	1.26E-07
MF0026	cysteine biosynthesis/homocysteine degradation	amino acid degradation	polar, uncharged amino acid degradation	0.00001935	0.0000683	0.000055	0.0000257	0.0000199	4.88E-06	5.19E-06
MF0027	cysteine degradation	amino acid degradation	polar, uncharged amino acid degradation	0.0003155	0.000472	0.000412	0.000114	0.000273	1.24E-13	4.09E-13
MF0028	serine degradation	amino acid degradation	polar, uncharged amino acid degradation	0.0009055	0.000912	0.001	0.00118268	0.000758	3.70E-09	5.16E-09
MF0029	threonine degradation (glycine pathway)	amino acid degradation	polar, uncharged amino acid degradation	0.00101	0.001112277	0.000718	0.0009	0.00072	2.90E-09	4.09E-09
MF0030	threonine degradation (formate pathway)	amino acid degradation	polar, uncharged amino acid degradation	0.001680946	0.0016	0.00153	0.00172	0.00123	7.13E-06	7.53E-06
MF0031	asparagine degradation	amino acid degradation	polar, uncharged amino acid degradation	0.0004705	0.00041	0.000603	0.000168	0.000348	4.58E-12	1.00E-11
MF0032	glutamine degradation (ammonia pathway)	amino acid degradation	polar, uncharged amino acid degradation	0.000186	0.0000776	0.00032	0.000661	0.000128	2.89E-14	1.42E-13
MF0033	cysteine degradation (mercaptopyruvate pathway)	amino acid degradation	polar, uncharged amino acid degradation	0.0003525	0.000289	0.000324	0.000579	0.000525	1.80E-11	3.42E-11
MF0034	glutamine degradation (oxoglutarate pathway)	amino acid degradation	polar, uncharged amino acid degradation	0.00125	0.00137605	0.00119	0.00193	0.00114	3.42E-14	1.55E-13
MF0035	arginine degradation (agmatine deiminase pathway)	amino acid degradation	positively charged amino acid degradation	0.000236	0.000182	0.000321	0.00102	0.000236	1.50E-11	2.90E-11
MF0036	arginine degradation (ornithine decarboxylase pathway)	amino acid degradation	positively charged amino acid degradation	0.00004685	0.000236	0.000374	0.0000258	0.0000236	6.84E-12	1.45E-11
MF0037	arginine degradation (AST/succinyltransferase pathway)	amino acid degradation	positively charged amino acid degradation	0.000018	0.00000746	0.000223	0.000013	0.0000051	3.89E-11	6.80E-11
MF0038	arginine degradation (arginine:pyruvate transaminase/4-aminobutyrate pathway)	amino acid degradation	positively charged amino acid degradation	0.000001095	0.00000136	0.0000135	0.000000594	0.00000054	7.32E-07	8.19E-07
MF0039	lysine fermentation to acetate and butyrate (3,6-diaminohexanoate pathway)	amino acid degradation	positively charged amino acid degradation	0.000176	0.000126	0.00011	0.0000608	0.0000871	0.0001	0.0001
MF0040	lysine degradation (cadaverine pathway)	amino acid degradation	positively charged amino acid degradation	0.000531	0.000552	0.000751	0.000198	0.000293	8.28E-09	1.08E-08
MF0041	histidine degradation	amino acid degradation	positively charged amino acid degradation	0.0001175	0.0000752	0.000243	0.0000654	0.0000646	8.38E-10	1.26E-09
MF0042	4-aminobutyrate degradation	amino acid degradation	positively charged amino acid degradation	0.000184	0.000208	0.000394	0.0000847	0.000158	8.02E-14	3.00E-13
MF0043	arginine degradation (agmatinase pathway)	amino acid degradation	positively charged amino acid degradation	0.0003375	0.000161	0.000382	0.000754	0.000406	1.89E-14	1.08E-13
MF0044	glycocholate degradation	bile acid degradation	glycocholate degradation	0.000109	0.000237	0.0000852	0.0000522	0.000178	5.39E-13	1.50E-12
MF0045	trehalose degradation	carbohydrate degradation	disaccharide degradation	0.000209	0.000176	0.0005	0.000061	0.000114	5.32E-16	1.11E-14
MF0046	sucrose degradation	carbohydrate degradation	disaccharide degradation	0.0005595	0.000745	0.000458	0.000481	0.000409	4.76E-12	1.02E-11
MF0047	lactose and galactose degradation (PTS)	carbohydrate degradation	disaccharide degradation	0.0000742	0.0000737	0.000122	0.0000238	0.0000394	2.86E-14	1.42E-13
MF0048	lactose degradation	carbohydrate degradation	disaccharide degradation	0.002285	0.002847678	0.00116	0.007282223	0.00222	9.25E-13	2.42E-12
MF0049	maltose degradation	carbohydrate degradation	disaccharide degradation	0.00009485	0.0000634	0.0000679	0.000107	0.0000559	5.74E-09	7.68E-09
MF0050	melibiose degradation	carbohydrate degradation	disaccharide degradation	0.001315	0.002266375	0.000585	0.00222	0.00119	1.84E-16	1.11E-14
MF0051	sucrose degradation (Actinobacteria)	carbohydrate degradation	disaccharide degradation	0.000553	0.000759	0.000447	0.000603	0.000426	4.27E-15	3.94E-14
MF0052	chondroitin sulfate and dermatan sulfate degradation	carbohydrate degradation	glycosaminoglycan degradation	0.000005395	0.00000285	0.0000808	0.00000208	0.00000162	1.58E-10	2.53E-10
MF0053	allose degradation	carbohydrate degradation	monosaccharide degradation	0.000173	0.000171	0.000123	0.000182	0.000116	1.62E-09	2.33E-09
MF0054	arabinose degradation	carbohydrate degradation	monosaccharide degradation	0.000263	0.000422	0.000382	0.0000839	0.000216	1.07E-14	7.39E-14
MF0055	xylose degradation	carbohydrate degradation	monosaccharide degradation	0.000553	0.00052	0.000519	0.00019	0.00039	8.34E-05	8.54E-05
MF0056	galactose degradation (Leloir pathway)	carbohydrate degradation	monosaccharide degradation	0.000658	0.000855	0.000451	0.000801	0.000639	3.32E-16	1.11E-14
MF0057	alpha-D-glucose and alpha-D-glucose 1-phosphate degradation	carbohydrate degradation	monosaccharide degradation	0.0003925	0.000487	0.000387	0.000487	0.000321	1.10E-13	3.85E-13
MF0058	fructose degradation	carbohydrate degradation	monosaccharide degradation	0.0005015	0.00056	0.00042	0.00013	0.000364	1.88E-11	3.53E-11
MF0059	rhamnose degradation	carbohydrate degradation	monosaccharide degradation	0.000276	0.000321	0.0003	0.000228	0.000269	1.15E-08	1.46E-08
MF0060	ribose degradation	carbohydrate degradation	monosaccharide degradation	0.0004625	0.000496	0.000527	0.000239	0.000921	8.14E-11	1.33E-10
MF0061	mannose degradation	carbohydrate degradation	monosaccharide degradation	0.0005335	0.000652	0.000413	0.000646	0.000416	1.18E-12	3.03E-12
MF0062	starch degradation	carbohydrate degradation	polysaccharide degradation	0.001068053	0.00123	0.000894	0.001092984	0.000718	9.74E-14	3.54E-13
MF0063	fructan degradation	carbohydrate degradation	polysaccharide degradation	0.000009135	0.0000178	0.00000377	0.00000244	0.00000519	1.30E-05	1.35E-05
MF0064	pectin degradation	carbohydrate degradation	polysaccharide degradation	0.00008105	0.0000682	0.000108	0.0000301	0.0000483	1.93E-05	1.99E-05
MF0065	pectin degradation - 5-dehydro-4-deoxy-D-glucuronate degradation	carbohydrate degradation	polysaccharide degradation	0.0002095	0.000184	0.000215	0.0000665	0.000127	1.10E-07	1.28E-07
MF0066	glycogen metabolism	carbohydrate degradation	storage	0.00134	0.00172	0.00101	0.00121	0.000966	3.60E-13	1.02E-12
MF0067	PHB production	carbohydrate degradation	storage	0.00000961	0.00000582	0.0000301	0.00000346	0.00000558	6.06E-11	1.03E-10
MF0068	glucarate degradation	carbohydrate degradation	sugar acid degradation	0.00008685	0.0000508	0.000182	0.0000231	0.0000567	3.14E-13	9.15E-13

Metabolic module ID	Metabolic module name	Hierarchy level 1	Hierarchy level 2	Prevotella-CAG	Lachnospiraceae-CAG	Pathogen-CAG	Akkermansia-Bacteroidales-CAG	Ruminococcaceae-CAG	p-value (Kruskal-Wallis)	q-value (Benjamini-Hochberg)
MF0069	galactarate degradation	carbohydrate degradation	sugar acid degradation	0.00007245	0.0000345	0.000182	0.0000191	0.0000402	2.00E-12	4.78E-12
MF0070	galactonate degradation	carbohydrate degradation	sugar acid degradation	0.00002695	0.0000211	0.000118	0.00000513	0.0000165	1.52E-12	3.82E-12
MF0071	D-galacturonate degradation	carbohydrate degradation	sugar acid degradation	0.00032	0.000313	0.000369	0.000106	0.000181	1.72E-08	2.12E-08
MF0072	ribitol degradation	carbohydrate degradation	sugar alcohol degradation	0.0002045	0.000208	0.00016	0.000212	0.000151	4.38E-10	6.67E-10
MF0073	sorbitol degradation (dehydrogenase)	carbohydrate degradation	sugar alcohol degradation	0.000175	0.000121	0.000184	0.0000517	0.000119	2.46E-10	3.84E-10
MF0074	mannitol degradation	carbohydrate degradation	sugar alcohol degradation	0.0001325	0.000212	0.00032	0.0000472	0.0000819	6.84E-16	1.12E-14
MF0075	sorbitol degradation (phosphotransferase)/sorbose degradation	carbohydrate degradation	sugar alcohol degradation	0.0000506	0.0000352	0.000154	0.00000803	0.0000296	1.96E-15	2.48E-14
MF0076	arabitol degradation	carbohydrate degradation	sugar alcohol degradation	0.000452	0.000416	0.000395	0.000149	0.000328	1.23E-05	1.29E-05
MF0077	galactitol degradation	carbohydrate degradation	sugar alcohol degradation	0.0002325	0.000222	0.000274	0.00021	0.000229	1.30E-11	2.58E-11
MF0078	xylitol degradation	carbohydrate degradation	sugar alcohol degradation	0.000447	0.000424	0.000368	0.00015	0.000333	2.22E-06	2.39E-06
MF0079	bifidobacterium shunt	central metabolism	energy metabolism	0.0005935	0.000716	0.000528	0.00055	0.000453	1.89E-12	4.59E-12
MF0080	Glycolysis (preparatory phase)	central metabolism	energy metabolism	0.001040836	0.001063713	0.000705	0.001383952	0.000854	1.25E-13	4.09E-13
MF0081	Glycolysis (pay-off phase)	central metabolism	energy metabolism	0.0009675	0.001087168	0.000739	0.001283599	0.000968	6.38E-15	4.92E-14
MF0082	pentose phosphate pathway (oxidative branch)	central metabolism	energy metabolism	0.0001755	0.00038	0.000407	0.0000738	0.00012	4.10E-14	1.79E-13
MF0083	pyruvate dehydrogenase complex	central metabolism	energy metabolism	0.0004895	0.000386	0.000767	0.0009	0.00047	1.67E-12	4.12E-12
MF0084	pyruvate:ferredoxin oxidoreductase	central metabolism	energy metabolism	0.0002	0.000148	0.0000566	0.0000795	0.000389	1.37E-14	8.56E-14
MF0085	pyruvate:formate lyase	central metabolism	energy metabolism	0.002566692	0.00245	0.002217619	0.002449205	0.001985651	8.92E-05	9.06E-05
MF0086	TCA cycle	central metabolism	energy metabolism	0.0005715	0.000574	0.000579	0.00115441	0.000637	8.88E-08	1.05E-07
MF0087	TCA cycle (Mycobacterium pathway)	central metabolism	energy metabolism	0.0004525	0.000474	0.000472	0.000737	0.000465	1.02E-06	1.14E-06
MF0088	TCA cycle (Helicobacter pathway)	central metabolism	energy metabolism	0.00055	0.000575	0.000499	0.000834	0.000579	7.40E-11	1.24E-10
MF0089	Entner-Doudoroff pathway I	central metabolism	energy metabolism	0.0001735	0.00014	0.000307	0.0000458	0.000106	7.57E-15	5.51E-14
MF0090	pentose phosphate pathway (non-oxidative branch)	central metabolism	energy metabolism	0.0008365	0.000917	0.000764	0.000807	0.000608	1.12E-13	3.85E-13
MF0091	beta-D-glucuronide and D-glucuronate degradation	glycoprotein degradation	glucuronide degradation	0.000324	0.000351	0.000378	0.000123	0.00019	2.22E-09	3.17E-09
MF0092	kdo2-lipid A synthesis	endotoxin biosynthesis	lipopolysaccharide biosynthesis	0.0001455	0.0000339	0.000281	0.000412	0.000092	2.92E-14	1.42E-13
MF0093	homoacetogenesis	gas metabolism	acetogenesis	0.0004655	0.000487	0.000231	0.000554	0.000403	6.22E-15	4.92E-14
MF0094	hydrogen metabolism	gas metabolism	hydrogen metabolism	0.0000671	0.0000863	0.0000247	0.000028	0.0000382	1.49E-09	2.17E-09
MF0095	NADH:ferredoxin oxidoreductase	gas metabolism	hydrogen metabolism	0.0000255	0.0000199	0.0000782	0.00000934	0.0000103	2.34E-13	7.30E-13
MF0096	methane oxidation	gas metabolism	methane consumption	0.000000124	4.12E-08	7.64E-08	2.75E-08	2.67E-08	0.0001	0.0001
MF0097	Methanogenesis - methyl-coM	gas metabolism	methanogenesis	0.000007325	0.000000873	0.00000165	0.000000164	0.00072	1.18E-09	1.74E-09
MF0098	methanogenesis from carbon dioxide	gas metabolism	methanogenesis	0.00000647	0.00000263	0.0000025	0.00000266	0.00044	6.18E-09	8.18E-09
MF0099	methanol conversion	gas metabolism	methanogenesis	0.00000848	0.0000026	0.00000269	0.00000124	0.000449	3.53E-10	5.44E-10
MF0100	Sulfate reduction (dissimilatory)	gas metabolism	sulfate metabolism	0.000118	0.0000485	0.000112	0.000251	0.0000847	2.08E-15	2.48E-14
MF0101	Sulfate reduction (assimilatory)	gas metabolism	sulfate metabolism	0.000194	0.0000626	0.00034	0.000724	0.000118	2.22E-14	1.21E-13
MF0102	mucin degradation	glycoprotein degradation	mucus degradation	0.0003685	0.000435	0.000221	0.00302	0.000456	1.15E-11	2.31E-11
MF0103	nitrate reduction (assimilatory)	inorganic nutrient metabolism	nitrogen	0.0008755	0.000851	0.000588	0.001195091	0.000886	1.30E-15	1.90E-14
MF0104	nitrate reduction (dissimilatory)	inorganic nutrient metabolism	nitrogen	0.0001245	0.0000753	0.00021	0.0000721	0.0000904	1.53E-08	1.91E-08
MF0105	nitrate reduction (denitrification)	inorganic nutrient metabolism	nitrogen	0.00004065	0.0000244	0.000106	0.0000155	0.0000306	3.03E-13	9.15E-13
MF0106	anaerobic fatty acid beta-oxidation	lipid degradation	beta-oxidation	0.0000671	0.0000374	0.000376	0.0000168	0.0000432	2.55E-11	4.65E-11
MF0107	glycerol degradation (propanediol pathway)	lipid degradation	glycerol degradation	0.0000154	0.0000214	0.0000111	0.00000513	0.0000144	3.84E-09	5.30E-09
MF0108	glycerol degradation (dihydroxyacetone pathway)	lipid degradation	glycerol degradation	0.0003155	0.000383	0.000293	0.000285	0.000326	4.91E-11	8.47E-11
MF0109	glycerol degradation (glycerol kinase pathway)	lipid degradation	glycerol degradation	0.0007705	0.000732	0.000708	0.000388	0.000569	4.45E-08	5.35E-08
MF0110	glyoxylate bypass	lipid degradation	glyoxylate bypass	0.000654	0.000673	0.000597	0.001110952	0.000625	5.27E-09	7.12E-09
MF0111	triacylglycerol degradation	lipid degradation	triacylglycerol degradation	0.00002545	0.0000192	0.000029	0.00000849	0.0000139	1.21E-07	1.39E-07
MF0112	acetate to acetyl-CoA	organic acid metabolism	acetate metabolism	0.0007965	0.000741	0.000747	0.000381	0.00216	2.30E-10	3.64E-10
MF0113	acetyl-CoA to acetate	organic acid metabolism	acetate metabolism	0.00076	0.000861	0.000503	0.000589	0.000616	8.40E-09	1.09E-08
MF0114	acetyl-CoA to crotonyl-CoA	organic acid metabolism	butyrate metabolism	0.0003245	0.000227	0.000546	0.000144	0.000322	4.43E-16	1.11E-14
MF0115	Crotonyl-coA from succinate	organic acid metabolism	butyrate metabolism	0.0001075	0.0000696	0.0000853	0.00019	0.000151	2.98E-15	3.01E-14
MF0116	butyrate production via transferase	organic acid metabolism	butyrate metabolism	0.0002465	0.000214	0.000119	0.0000832	0.000124	1.64E-06	1.78E-06
MF0117	butyrate production via kinase	organic acid metabolism	butyrate metabolism	0.000232	0.000201	0.000105	0.0000835	0.000181	1.27E-07	1.45E-07
MF0118	formate conversion	organic acid metabolism	formate metabolism	0.0002335	0.00016	0.000419	0.0000447	0.000435	1.52E-14	9.08E-14
MF0119	lactate production	organic acid metabolism	lactate metabolism	0.0006845	0.000992	0.000424	0.000821	0.000509	1.31E-14	8.56E-14
MF0120	lactate consumption	organic acid metabolism	lactate metabolism	0.00006355	0.0000197	0.000259	0.0000218	0.0000259	4.16E-12	9.23E-12
MF0121	propionate production (acrylate pathway)	organic acid metabolism	propionate metabolism	0.000111	0.0000591	0.000145	0.0000384	0.000059	3.36E-11	5.95E-11
MF0122	propionate production (succinate pathway)	organic acid metabolism	propionate metabolism	0.000149	0.0000812	0.000155	0.00099	0.000205	1.38E-10	2.24E-10
MF0123	propionate production (propanediol pathway)	organic acid metabolism	propionate metabolism	0.0001075	0.000145	0.000124	0.000032	0.0000811	7.80E-12	1.62E-11
MF0124	Fucose degradation	organic acid metabolism	propionate metabolism	0.000269	0.00023	0.000249	0.000702	0.000306	1.04E-09	1.54E-09
MF0125	propionate production via kinase	organic acid metabolism	propionate metabolism	0.0000685	0.000158	0.000348	0.0000262	0.0000503	2.40E-11	4.43E-11
MF0126	propionate production via transferase	organic acid metabolism	propionate metabolism	0.0001265	0.0000772	0.000126	0.0000378	0.0000621	1.37E-06	1.51E-06
MF0127	Succinate production	organic acid metabolism	succinate metabolism	0.000432	0.000406	0.000485	0.001025394	0.000585	8.06E-11	1.33E-10
MF0128	Propionate conversion to succinate	organic acid metabolism	succinate metabolism	0.0000258	0.0000101	0.000235	0.0000077	0.00000838	3.03E-11	5.44E-11
MF0129	catalase	protection against oxidative stress	catalase	0.0003185	0.000153	0.000629	0.001524391	0.00029	9.94E-12	2.03E-11
MF0130	peroxidase	protection against oxidative stress	peroxidase	0.0004295	0.000426	0.000452	0.000856	0.000453	2.87E-08	3.51E-08
MF0131	superoxide dismutase	protection against oxidative stress	superoxide dismutase	0.000285	0.000184	0.000351	0.000495	0.000309	6.11E-14	2.50E-13
MF0132	superoxide reductase	protection against oxidative stress	superoxide reductase	0.000112	0.000109	0.0000336	0.0000439	0.000169	2.51E-15	2.74E-14
MF0133	menaquinone production	secondary metabolites	vitamins	0.0002065	0.000168	0.00026	0.00045	0.000203	1.34E-11	2.63E-11

Abundances of inferred metabolic modules for each co-abundance group (CAG) of microbes in the subset of participants forming high-abundance poles (HAPs; n=114). P-values (from Kruskal-Wallis tests) and q-values (Benjamini-Hochberg correction) denote differences in the functional potential among CAGs.

Table S7. Correlations between relevant metabolic modules and co-abundance groups (CAGs) of microbes in the complete dataset.

	Prevotella-CAG		Lachnospiraceae-CAG		Pathogen-CAG		Akkermansia-Bacteroidales-CAG		Ruminococcaceae-CAG	
**Modules Figure 5A**	Rho	q value	Rho	q value	Rho	q value	Rho	q value	Rho	q value
Lipopolysaccharide biosynthesis	0.02	0.06	-0.17	0.05	0.50	0.0002	-0.03	<0.0001	-0.41	<0.0001
Mucus degradation	-0.18	<0.0001	0.35	<0.0001	-0.09	<0.0001	0.33	<0.0001	-0.49	0.04
Methanogenesis	0.20	<0.0001	-0.15	<0.0001	-0.13	<0.0001	-0.27	<0.0001	0.58	0.004

	Prevotella-CAG		Lachnospiraceae-CAG		Pathogen-CAG		Akkermansia-Bacteroidales-CAG		Ruminococcaceae-CAG	
**Modules Figure 5B**	Rho	q value	Rho	q value	Rho	q value	Rho	q value	Rho	q value
Lipopolysaccharide biosynthesis	0.21	<0.0001	0.15	<0.0001	-0.18	0.00002	-0.34	<0.0001	0.41	<0.0001
Mucus degradation	0.13	<0.0001	0.25	<0.0001	0.29	<0.0001	-0.37	0.04	-0.26	<0.0001
Methanogenesis	-0.24	0.0002	-0.25	<0.0001	0.14	0.0003	0.47	0.04	-0.31	0.03

	Prevotella-CAG		Lachnospiraceae-CAG		Pathogen-CAG		Akkermansia-Bacteroidales-CAG		Ruminococcaceae-CAG	
**Modules Figure 5C**	Rho	q value	Rho	q value	Rho	q value	Rho	q value	Rho	q value
Lipopolysaccharide biosynthesis	0.41	<0.0001	0.07	<0.0001	0.29	<0.0001	-0.67	0.01	0.01	0.09
Mucus degradation	-0.05	0.006	-0.73	0.001	0.05	<0.0001	0.19	<0.0001	0.24	<0.0001
Methanogenesis	-0.05	0.0008	0.70	<0.0001	-0.10	<0.0001	-0.05	<0.0001	-0.24	<0.0001

Correlations between the relative abundances of metabolic modules depicted in Fig. 5 and CAG abundances in the complete dataset (n=441). Spearman's rho and q-values are shown.
